# A *Vibrio cholerae* anti-phage system depletes nicotinamide adenine dinucleotide to restrict virulent bacteriophages

**DOI:** 10.1128/mbio.02457-24

**Published:** 2024-10-08

**Authors:** Yishak A. Woldetsadik, David W. Lazinski, Andrew Camilli

**Affiliations:** 1 Department of Molecular Biology and Microbiology, Graduate School of Biomedical Sciences, Tufts University School of Medicine, Boston, Massachusetts, USA; University of Pittsburgh, Pittsburgh, Pennsylvania, USA

**Keywords:** *Vibrio cholerae*, bacteriophages, bacteriophage evolution, cholera, bacteriophage immunity

## Abstract

Bacteria and their predatory viruses (bacteriophages or phages) are in a perpetual molecular arms race. This has led to the evolution of numerous phage defensive systems in bacteria that are still being discovered, as well as numerous ways of interference or circumvention on the part of phages. Here, we identify a unique molecular battle between the classical biotype of *Vibrio cholerae* and virulent phages ICP1, ICP2, and ICP3. We show that classical biotype strains resist almost all isolates of these phages due to a 25-kb genomic island harboring several putative anti-phage systems. We observed that one of these systems, Nezha, encoding SIR2*-*like and helicase proteins, inhibited the replication of all three phages. Bacterial SIR2-like enzymes degrade the essential metabolic coenzyme nicotinamide adenine dinucleotide (NAD^+^), thereby preventing replication of the invading phage. In support of this mechanism, we identified one phage isolate, ICP1_2001, which circumvents Nezha by encoding two putative NAD^+^ regeneration enzymes. By restoring the NAD^+^ pool, we hypothesize that this system antagonizes Nezha without directly interacting with its proteins and should be able to antagonize other anti-phage systems that deplete NAD^+^.

**IMPORTANCE:**

Bacteria and phages are in a perpetual molecular arms race, with bacteria evolving an extensive arsenal of anti-phage systems and phages evolving mechanisms to overcome these systems. This study identifies a previously uncharacterized facet of the arms race between *Vibrio cholerae* and its phages. We identify an NAD^+^-depleting anti-phage defensive system called Nezha, potent against three virulent phages. Remarkably, one phage encodes proteins that regenerate NAD^+^ to counter the effects of Nezha. Without Nezha, the NAD^+^ regeneration genes are detrimental to the phage. Our study provides new insight into the co-evolutionary dynamics between bacteria and phages and informs the microbial ecology and phage therapy fields.

## INTRODUCTION

*Vibrio cholerae* is a Gram-negative facultative pathogen that resides in estuarine environments and causes the severe diarrheal disease cholera. There are two biotypes of *V. cholerae*: classical, which caused the first six recorded cholera pandemics, and El Tor, responsible for the ongoing 7th pandemic (1). In areas surrounding the Bay of Bengal, *V. cholerae* is frequently preyed upon within the intestinal tract of cholera victims by three virulent phages: ICP1, ICP2, and ICP3. ICP1 is the most commonly isolated of the three phages in cholera patient stools and the aquatic environment (2, 3) and is the only one that can prey on *V. cholerae* in the aquatic environment (4). Thus, *V. cholerae* and ICP1 frequently engage in a co-evolutionary arms race. As a result, *V. cholerae* possesses several lines of defense against ICP1, some appearing universal in the species and some varying between strains. One universal mechanism is the phase variation of the lipopolysaccharide O1-antigen, which is the receptor for ICP1 (5). Other defense systems are cytoplasmically located and include diverse mechanisms that interfere with the ICP1 life cycle, such as phage-inducible chromosomal island-like elements (PLEs) present in many classical and El Tor strains and the cyclic-oligonucleotide-based anti-phage signaling system (CBASS) found in all El Tor strains (6, 7).

The PLE excises from the chromosome upon ICP1 infection and blocks phage replication by degrading the phage DNA and causing abortive infection by premature lysis. PLEs can spread horizontally by hijacking ICP1 structural components to make PLE-transducing virions (8). Some ICP1 isolates overcome this potent defense system by encoding a Type I-F CRISPR/Cas system or a standalone nuclease, both targeting the PLE (9). Classical biotype strains appear to have only one version of the PLE, PLE5, whereas El Tor strains may have one of several different PLE types, though not PLE5 (10). In addition, the El Tor biotype lacks a CRISPR/Cas system, whereas classical strains encode a Type 1-E CRISPR/Cas system for phage defense (11, 12). Given the differences in phage defensive systems between classical and El Tor biotypes, we asked whether there are biotype differences in sensitivity to ICP1 infection. Here, we use a collection of classical biotype strains and ICP1 isolates to show that classical biotype strains are almost universally resistant to ICP1 independent of CRISPR/Cas. Based on this, we hypothesized that classical biotype strains encode an unknown anti-phage system that restricts ICP1.

To uncover this anti-phage system, we used whole-genome sequencing and bioinformatics to identify two putative anti-phage systems, Gabija and Nezha, residing on a 25-kb genetic element unique to the classical biotype. These two systems were recently characterized in other bacteria. Gabija encodes for a protein complex composed of a GajA nuclease and GajB helicase (13, 14). Gabija is activated by NTP depletion, resulting in the selective degradation of DNA in phage-infected cells (15). Nezha encodes for a protein complex composed of a SIR2-like nicotinamide adenine dinucleotide (NAD^+^) hydrolase and a HerA helicase (16–18). Nezha depletes the essential metabolic coenzyme NAD^+^, thus blocking phage replication (16, 17, 19). Here, we show Nezha is responsible for conferring resistance to ICP1, ICP2, and ICP3. Highlighting the consequences of phage-host coevolution, we identify one ICP1 isolate that counters Nezha using an NAD^+^ regenerating system. Our results provide insight into a previously uncharacterized host-phage arms race in classical biotype *V. cholerae*.

## RESULTS

### Classical biotype *V. cholerae* encodes anti-phage systems absent in the El Tor biotype

**Fig 1:**
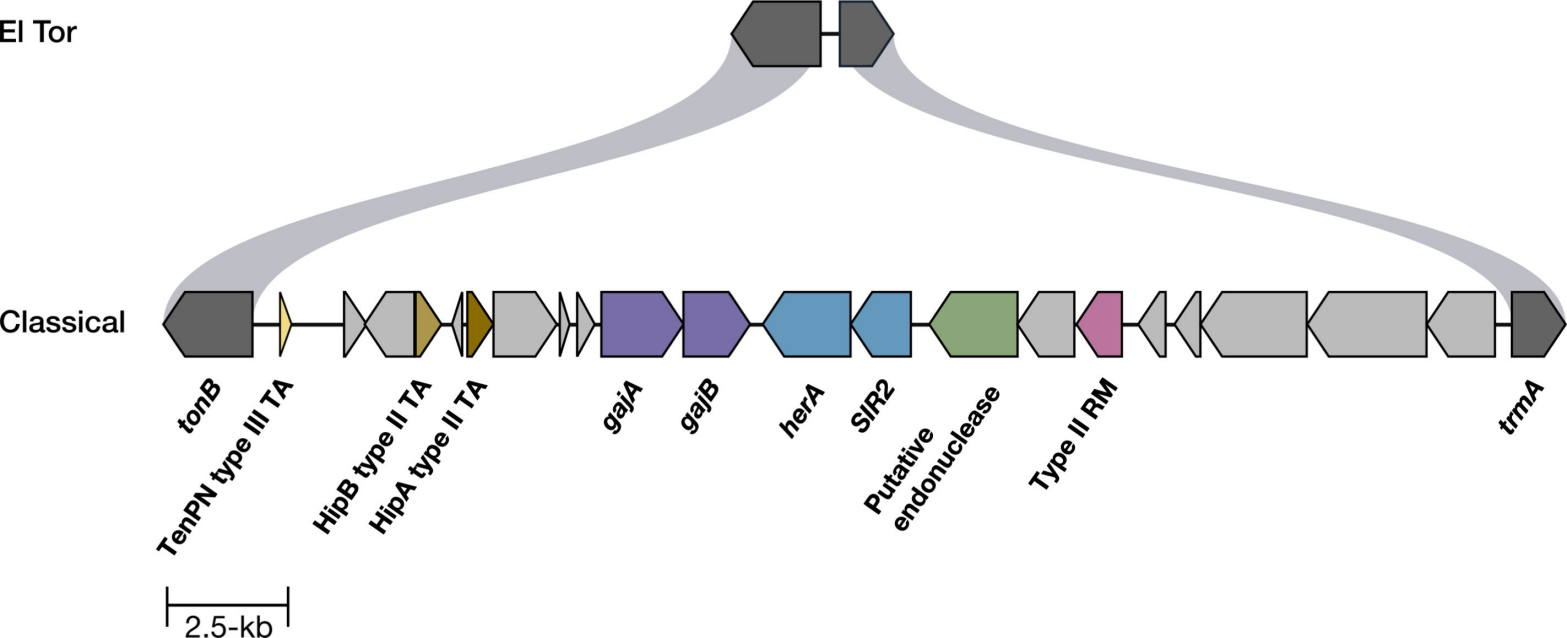
Classical biotype *V. cholerae* encodes a genomic island with phage immunity genes. Schematic showing the genome organization of a unique 25-kb genomic island flanked by *tonB* and *trmA* in classical *V. cholerae* strain A103. From left to right, the island contains several putative anti-phage systems, including a type III toxin-antitoxin system, type II toxin-antitoxin system, Gabija (*gajAB*), Nezha (*SIR2-herA*), an endonuclease, and a type II restriction-modification system. Genes in gray are of unknown function.

Using a collection of 12 classical biotype *V. cholerae* clinical strains isolated from multiple countries between 1949 and 1970, we found that all were resistant to infection by the modern virulent *Myoviridae* phage ICP1_2011_A. By contrast, eight El Tor biotype clinical isolates from 1978 to 2022 were sensitive to this phage. Phage sensitivity was assessed by plaque assay on LB soft agar overlays at 37°C. Although we lack ICP1 isolates from 1949 to 1970, we infer this phage was present due to the ICP1-specific PLE5 immunity system in classical biotype strains (10). ICP1_2011_A blocks PLE5 immunity by encoding a CRISPR/Cas system with a spacer targeting PLE5 (9). To further probe this biotype difference in phage resistance, we tested representative classical and El Tor biotype strains A103 and E7946, respectively, for sensitivity to a panel of ICP1 isolates from 2001 to 2011. A103 was resistant to all ICP1 phages except one, ICP1_2001, whereas E7946 was sensitive to all (Table 1; Fig. S1). Based on this, we hypothesize that classical biotype *V. cholerae* possesses a phage defensive system absent from El Tor and that ICP1_2001 contains gene(s) absent from the other ICP1 isolates that allow it to overcome the phage defensive system. We used whole-genome sequencing and bioinformatic analysis to identify differences between the two *V. cholerae* biotypes concerning phage resistance. Putative anti-phage systems were identified using the program DefenseFinder (20–22). We found a 25-kb genomic island in all 12 classical biotype strains that were absent in El Tor, located between the common genes *tonB* and *trmA* (Fig. 1). This island encodes several putative anti-phage systems, including Gabija (*gajAB*) and Nezha (*SIR2-herA*). Based on this, we hypothesized that Gabija and/or Nezha contribute to the phage resistance observed for classical biotype strains.

**TABLE 1:** Phage sensitivity of representative *Vibrio cholerae* El Tor and classical biotype strains against ICP1 phage isolates^*a*^

Strain	ICP1_2001	ICP1_2001_A	ICP1_2004_A	ICP1_2005_A	ICP1_2006_C	ICP1_2006_D	ICP1_2006_E	ICP1_2011_A
E7946 El Tor	+	+	+	+	+	+	+	+
A103 classical	+	−	−	−	−	−	−	−

^*a*^ + indicates sensitivity, while − indicates resistance to the phage isolate.

### Nezha provides immunity against ICP1

**Fig 2:**
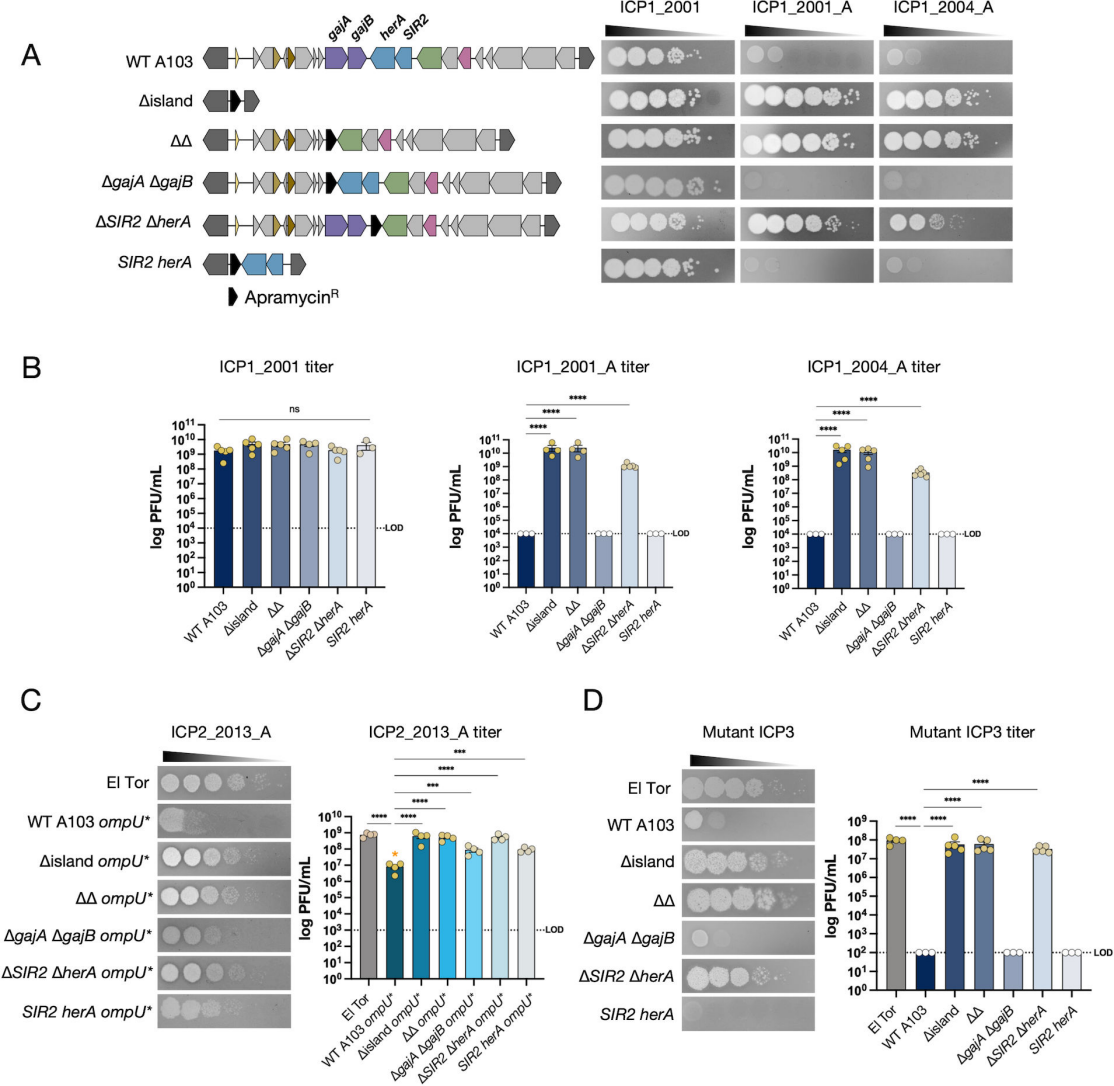
SIR2-HerA protects classical biotype *V. cholerae* from several phages. (**A**) Schematic of the gene deletions constructed in the classical biotype strain A103 (left) and ICP1 phage sensitivity profiles (right). The phage sensitivity of each strain on the left is shown by plaque formation after spotting 10-fold serial dilutions of the ICP1 phages indicated at the top. ICP1_2001 and ICP1_2001_A are distinct isolates from the same year. The results from three to five biological replicate experiments are quantified in (**B**). White-filled circles indicate titers below the limit of detection (LOD) indicated by the horizontal dashed lines. (**C**) Phage sensitivity of the strains indicated to phage ICP2_2013_A (left) and quantification of the results from four biological replicates (right). El Tor is strain E7946; the rest are classical strain A103 wild type and derivatives. Because the gene for the ICP2 receptor, *ompU*, is naturally mutated in A103, it was replaced with the El Tor E7946 *ompU* (indicated by ompU*). The orange asterisk (*) indicates incubation for 3 hours at 37°C. (**D**) Phage sensitivity of the strains indicated to mutant ICP3 phage (left) and quantification of the results from three to five biological replicates (right). Data are shown as the standard error of the mean. Analysis was performed using one-way ANOVA with Dunnett’s multiple comparison test (*P < 0.05, **P < 0.01, ***P < 0.001, ****P < 0.0001).

We generated marked gene deletions using an apramycin resistance cassette to test the role of the whole island, Gabija, and Nezha in the classical biotype strain A103. Five deletion strains were made: ∆island, a double system deletion ∆∆ (∆*gajA* ∆*gajB* and ∆*SIR2* ∆*herA*)*,* the single system deletions (∆*gajA* ∆*gajB* or ∆*SIR2* ∆*herA*), and deletion of all genes in the island except for *SIR2 herA* (Fig. 2A). These and other mutants constructed in this study were whole genome sequenced to confirm the desired mutations and lack of other mutations. The role of Gabija or Nezha in phage resistance was tested by plaque assays on the wild type or mutant A103 derivatives using soft agar overlays containing individual *V. cholerae* strains and spotting 10-fold serial dilutions of the phages indicated. As expected, the wild-type A103 strain was sensitive to ICP1_2001 but resistant to ICP1_2001_A and ICP1_2004_A (Fig. 2A and B). However, the ∆island and the ∆∆ mutants were sensitive to all three phages, suggesting that Gabija and/or Nezha mediate phage resistance. Assaying the single system deletion strains revealed that Nezha (*SIR2 herA*) is the primary anti-phage system and that Gabija plays only a minor role in resistance. This was confirmed by showing that deletion of the entire island except for *SIR2 herA* retained resistance to ICP1_2001_A and ICP1_2004_A (Fig. 2A and B). Overall, these results show that most ICP1 isolates are blocked by Nezha.

### Nezha provides immunity against ICP2 and ICP3

We tested whether Nezha protects against other phages. In contrast to ICP1 and the *Podoviridae* phage ICP3, which both use the lipopolysaccharide O1-antigen as their receptors (2, 23), the distinct *Podoviridae* phage ICP2 uses the outer membrane porin OmpU as its receptor (24). Work done using the El Tor biotype showed that amino acid changes at positions V324 and G325 within an outer loop of OmpU confer resistance to ICP2 (24, 25). In the classical strain A103, OmpU naturally varies at both positions (V324A and G325S), leading to ICP2 resistance in both the wild-type and ∆island strains (Fig. S2). To overcome this block, we transformed naturally competent A103 and its derivatives to replace *ompU* with that from El Tor strain E7946 (designated ompU* in Fig. 2C). Classical strain A103 with El Tor *ompU* (WT OmpU*) was partially sensitive to ICP2, as small plaques formed after 3 hours at 37°C at the two highest phage concentrations but then disappeared after 4 hours. By contrast, infecting both ∆island OmpU* and ∆∆ OmpU* strains yielded complete sensitivity to ICP2 with plaquing efficiencies comparable to that on the El Tor E7946-positive control strain (Fig. 2C). Once again, Nezha appears to be the primary resistance determinant, as shown by a significant 100-fold increase in plaquing efficiency in ∆*SIR2* ∆*herA* OmpU* compared to WT OmpU*, which was comparable to the plaquing efficiencies on the ∆island OmpU* and ∆∆ OmpU* strains.

Classical strain A103 was also resistant to infection by ICP3 (Fig. 2D). We do not believe this resistance is due to receptor availability since the classical and El Tor biotypes express the same O1-antigen. In contrast to ICP1 and ICP2, deleting Nezha and Gabija or the entire island did not allow for plaque formation by ICP3. We hypothesized that classical strain A103 has an additional phage defensive system against ICP3 that obscures Nezha and Gabija’s potential role. To circumvent this putative additional ICP3 defense system, we sought to isolate spontaneous mutants of ICP3 that could form plaques on the ∆island strain. We readily obtained ICP3 mutants that formed plaques on the A103 ∆island strain. Sequencing two independent pairs of ICP3 mutants revealed that each pair had a frameshift mutation in different locations within *orf17* (Table S1). Orf17 lacks detectable sequence or predicted structural homology to proteins of known function, so the nature of the host immune mechanism circumvented by these null mutations is unknown. Regardless, the mutant ICP3 allowed us to probe the roles of Nezha and Gabija. Upon infecting a ∆*gajA* ∆*gajB* strain*,* we observed no plaques, which suggests that Gabija does not inhibit ICP3. However, infection of a ∆*SIR2* ∆*herA* strain rescued plaque formation to a level equivalent to that on the ∆island and El Tor E7946 strains (Fig. 2D). This indicates that Nezha, but not Gabija, antagonizes ICP3. These data show that three different phages, ICP1, ICP2, and ICP3, are antagonized by the Nezha (*SIR2 herA*) anti-phage system, with Gabija (*gajAB*) playing a minor role in resistance to ICP1_2001_A, ICP1_2004_A, and ICP2.

### Mutating the active site of SIR2 or deleting *herA* abolishes phage immunity

**Fig 3:**
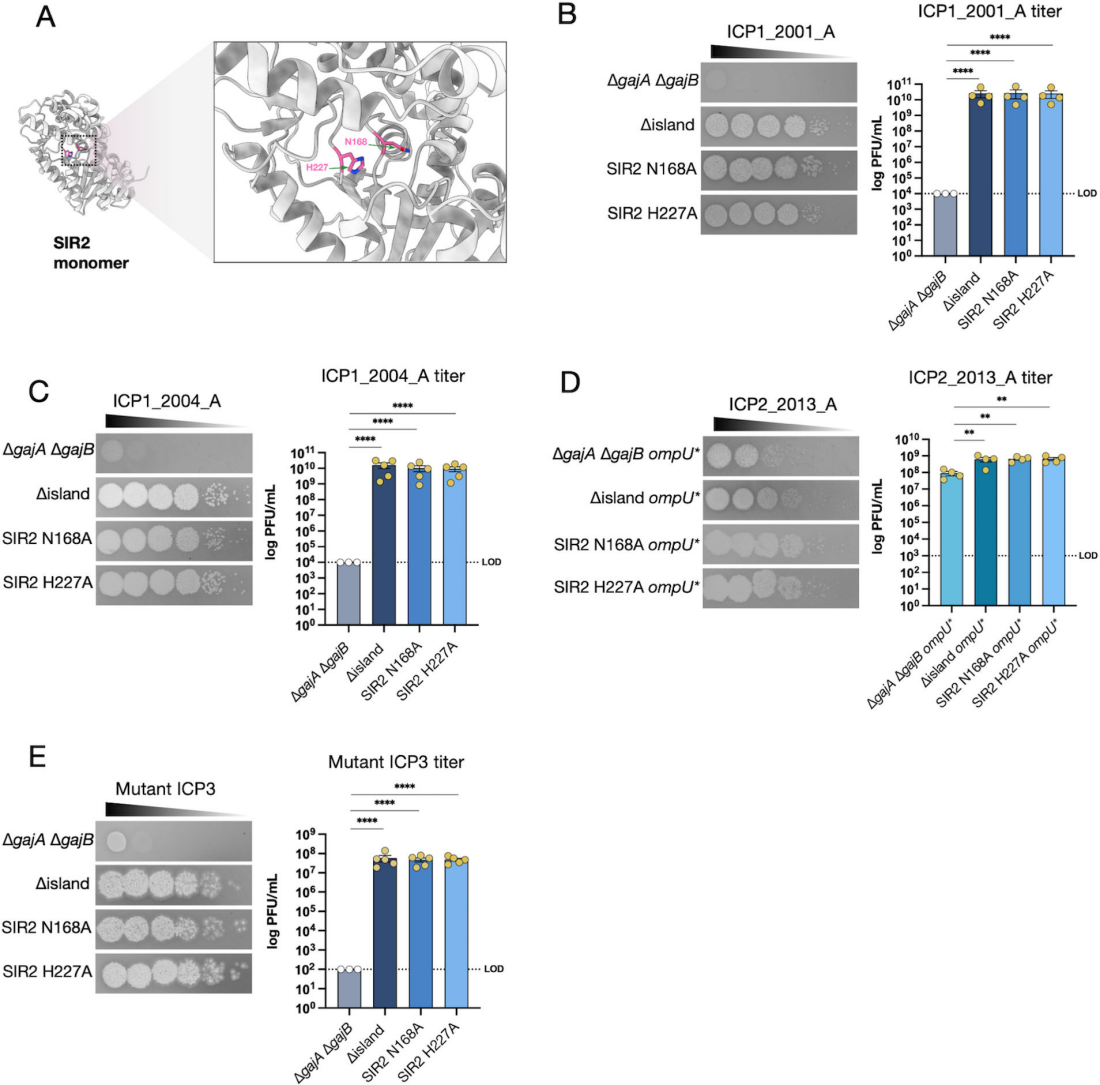
Mutation of the SIR2 NADase active site abolishes immunity to ICP1, ICP2, and ICP3. (**A**) The SIR2 protein monomer’s structure predicted by Alphafold (18) shows active site residues N168A and H227A (16). (**B and C**) Phage sensitivity of the strains indicated to ICP1 phages (left) and quantification of the results from three to five biological replicates (right). (**D**) Phage sensitivity of the strains indicated to ICP2 phage (left) and quantification of the results from four biological replicates (right). (**E**) Phage sensitivity of the strains indicated to the mutant ICP3 phage (left) and quantification of the results from three to five biological replicates (right). Data are shown as the standard error of the mean. Analysis was performed using one-way ANOVA with Dunnett’s multiple comparison test (*P < 0.05, **P < 0.01, ***P < 0.001, ****P < 0.0001).

The Nezha homologs SIR2 and HerA from *Escherichia coli* form a large protein complex that provides phage immunity through multiple mechanisms, including NAD^+^ hydrolysis, helicase, and nuclease activities (16, 17). However, other studies on homologous systems showed that point mutations in the conserved NADase active site of SIR2 are sufficient to abolish immunity, suggesting that depletion of NAD^+^ is critical for Nezha-mediated phage defense (16, 17, 19). Amino acid sequence and protein structure alignments to *E. coli* SIR2 indicate that N168 and H227 of A103 SIR2 are conserved NADase active site residues (Fig. 3A). To examine whether the NADase activity of the A103 SIR2 protein is important for phage defense, we generated alanine substitutions in both active site residues in the ∆*gajA* ∆*gajB* mutant strain background (Fig. 3A). Active site mutations N168A or H227A abolished immunity to ICP1 2001_A and 2004_A, ICP2, and ICP3 (Fig. 3B through F). Since HerA forms a complex with SIR2, it may be needed for SIR2 function. To test this, we deleted *herA* and measured phage sensitivity. We observed that ICP1_2001_A, ICP1_2004_A, and ICP3 had significantly higher plaquing efficiencies on the ∆*herA* strain when compared to WT A103, indicating that HerA complex formation with SIR2 is required for phage immunity (Fig. S3).

### ICP1_2001 encodes proteins that antagonize Nezha

**Fig 4:**
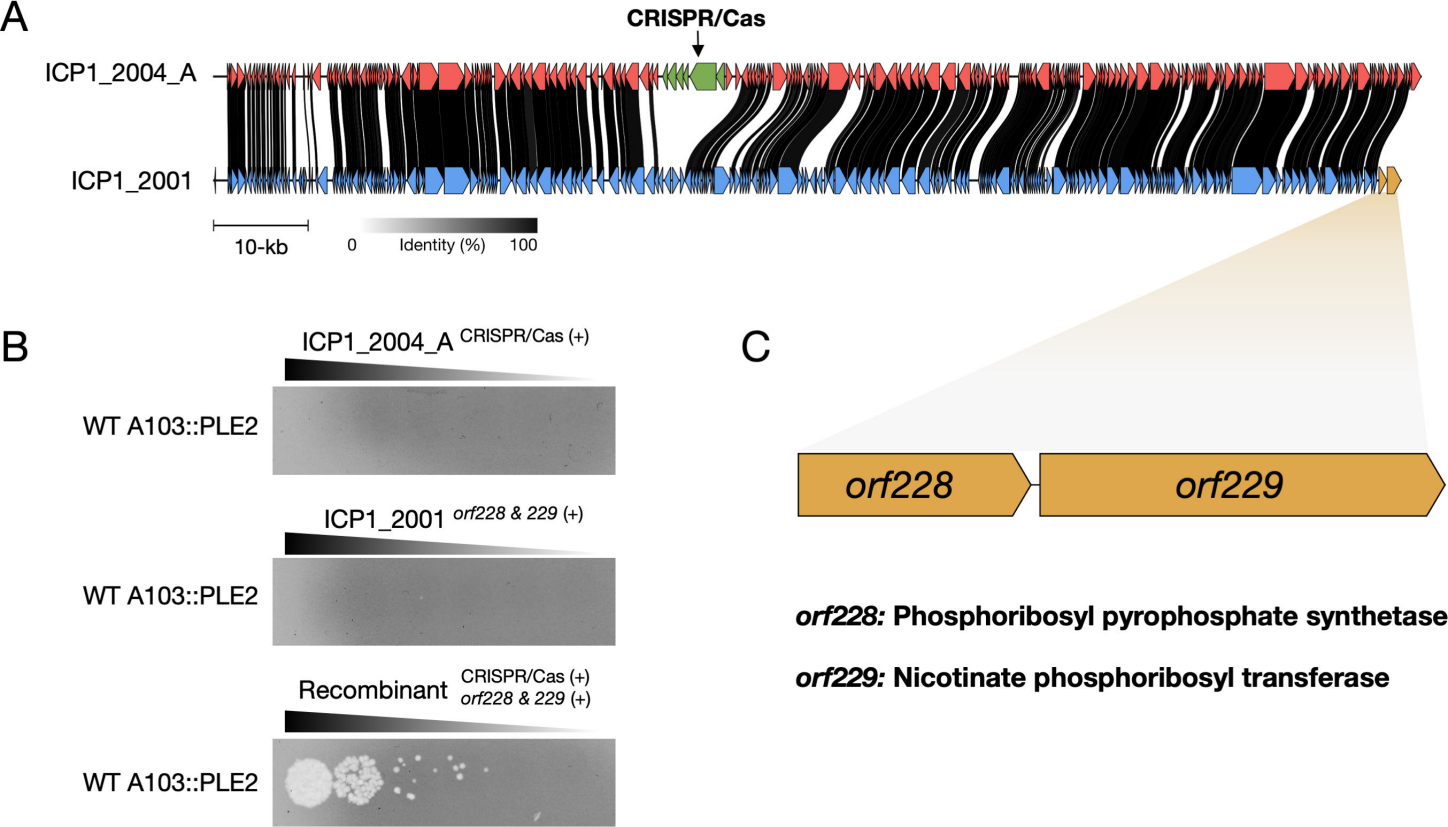
ICP1_2001 encodes for anti-Nezha proteins that overcome NAD^+^ depletion. (**A**) Genome comparison between ICP1_2001 (blue) and ICP1_2004_A (red). Homologous genes are connected with the black lines, where black lines indicate a higher percentage of identity. The CRISPR/Cas system of ICP1_2004_A is shown in green while the anti-Nezha genes of ICP1_2001 are shown in orange. (**B**) Phage sensitivity of classical biotype strain A103::PLE2 to both parent phages and a representative selected recombinant phage. The genotype of the phages is shown. (**C**) Schematic of the anti-Nezha genes encoded by ICP1_2001 and their annotation of the proteins they encode for.

The ICP1 phages used in this study differ across their ~130 kb genomes by many single nucleotide polymorphisms (SNPs) and a large number of unique regions, making it challenging to identify the genetic difference in ICP1_2001 that counters Nezha. To map the region responsible, we used a phage recombination experimental approach between ICP1_2001 and ICP1_2004_A. ICP1_2004_A was selected because it contains a CRISPR/Cas system with spacers targeting PLE2, thus providing a selection for recombinants of this phage. We reasoned that only recombinant phages that acquire CRISPR/Cas from ICP1_2004_A and the anti-Nezha locus from ICP1_2001 could plaque on a classical A103 derivative modified to contain PLE2. We generated a PLE2 transducing phage to move PLE2 tagged with a Spectinomycin resistance cassette into wild-type A103, ∆island*,* and ∆*SIR2* ∆*herA* derivatives. After isolating transductants, we confirmed that they had the correct phage sensitivity profiles: A103 PLE2 was resistant to both parental phages, while the ∆island PLE2 and ∆*SIR2* ∆*herA* PLE2 strains were resistant to ICP1_2001 but sensitive ICP1_2004_A, showing that PLE2 was functional against ICP1_2001 (Fig. S4). We generated seven independent recombinant phage pools by co-infecting the permissive El Tor strain E7946 with a 1:1 mixture of ICP1_2001 and ICP1_2004_A at a high multiplicity of infection (MOI) of 5. Recombinant phages that could plaque on the A103 PLE2 host were readily obtained from each pool (example shown in Fig. 4B). Two phages from each selection were plaque-purified on the A103 PLE2 host and subjected to whole-genome sequencing. As expected, mapping the sequencing reads to the ICP1_2004_A parent genome showed the presence of CRISPR/Cas with spacers against PLE2 in all 14 isolates. Conversely, mapping the sequencing reads to the ICP1_2001 parent genome showed the presence of the two gene locus located at the 3′ end of the genome in all 14 isolates. The genes in this putative two-gene operon, *orf228* and *orf229,* are annotated as phosphoribosyl pyrophosphate synthetase (PRS) and nicotinate phosphoribosyl transferase (NAPRT), respectively (Fig. 4C). It has been shown that PRS uses ribose-5-phosphate and ATP to generate phosphoribosyl pyrophosphate (PRPP). PRPP is utilized by several biosynthetic enzymes, including NAPRT, to generate nicotinic acid mononucleotide, one of several primary substrates that can participate in the synthesis of NAD^+^ (26). Based on Nezha’s anti-phage activity mechanism, we reasoned that ORF228 and ORF229 participate in the regeneration of NAD^+^ within the cell to counter Nezha.

### The ICP1_2001 NAD^+^ regenerating proteins are required to subvert Nezha

**Fig 5:**
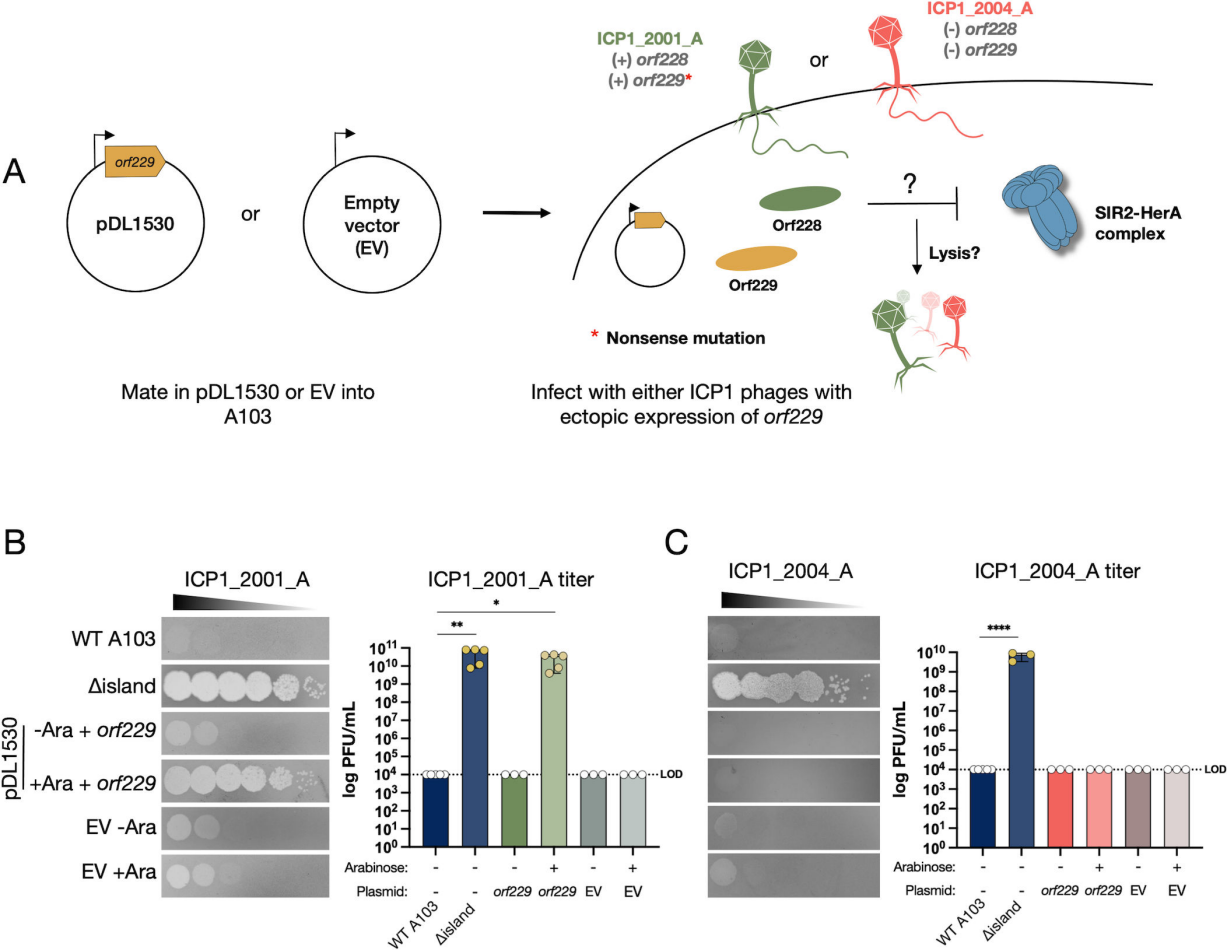
Expressing *orf229 in trans* rescues ICP1_2001_A but not ICP1_2004_A. (**A**) Cartoon schematic of the experimental outline to test the outcome of *orf229* ectopic expression during phage infection with ICP1_2001_A (green) and ICP1_2004_A (red). The genotypes of both phages are shown. (**B**) Phage sensitivity of the strains indicated to ICP1_2001_A or ICP1_2004_A. (**C**) Quantification of the results from three to five biological replicates is shown to the right in each panel. Expression of *orf229* from the plasmid was induced by adding arabinose (Ara) to the soft agar overlays. Data are shown as the standard error of the mean for parametric data or as the median with range for non-parametric data. Analysis was performed using one-way ANOVA with Dunnett’s multiple comparison test or Kruskal-Wallis with Dunn’s multiple comparison test (*P < 0.05, **P < 0.01, ***P < 0.001, ****P < 0.0001).

After uncovering the potential anti-Nezha genes of ICP1_2001, we wanted to see whether other Nezha-sensitive ICP1 isolates lack these two genes. Surprisingly, a Nezha-sensitive isolate, ICP1_2001_A, encodes versions of *orf228* and *orf229*. However, comparing their amino acid sequences reveals that the ICP1_2001_A ORF228 has two amino acid substitutions, V240I and E254G, and ORF229 has an E75G amino acid substitution and a nonsense mutation near the N-terminus (Y29*) that truncates the protein, presumably ablating its function in NAD^+^ regeneration. To test whether this defect can be bypassed, we cloned a functional *orf229* from ICP1_2001 into an arabinose inducible expression plasmid, pDL1530, such that ORF229 production is under the control of the P_BAD_ promoter. An empty vector (EV) was included as a control (Fig. 5A). After mating the plasmids into A103, we induced expression of ORF229 by adding arabinose to the soft agar overlay used for plaque assay. We used wild-type A103 and *∆*island as controls to show that ICP1_2001_A can only form plaques on the ∆island strain (Fig. 5B). Without arabinose, ICP1_2001_A could not form plaques on A103 (pDL1530::*orf229*). However, in the presence of arabinose, we observed complete rescue of ICP1_2001_A plaque formation, with comparable plaquing efficiency on the ∆island strain. As expected, the empty vector control strains were resistant to the phage. This result indicates that an intact *orf229* is needed to counter Nezha and suggests that the variant *orf228* allele in ICP1_2001_A is functional if it plays a role in countering Nezha. To confirm the role of *orf228* in countering Nezha, we did the same experiment using ICP1_2004_A as the infecting phage, which lacks *orf228* and *orf229*. ICP1_2004_A could not form plaques on the A103 strain expressing only *orf229* (Fig. 5C). This indicates that ORF228 is needed in conjunction with ORF229 to counter Nezha.

**Fig 6:**
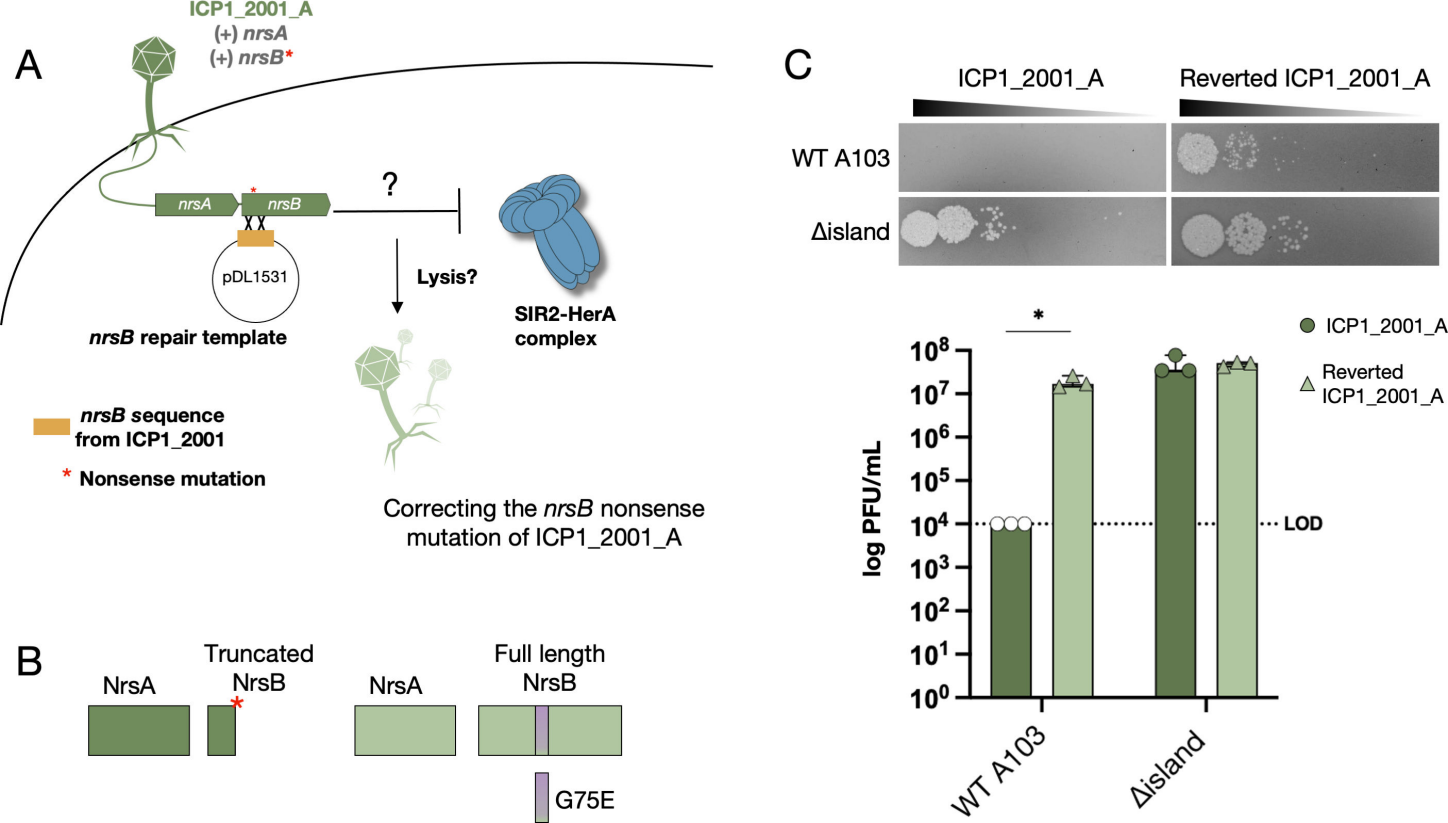
Reverting the nonsense mutation in *nrsB* rescues ICP1_2001_A infectivity. (**A**) Cartoon schematic of the experiment to revert the nonsense mutation. (**B**) Cartoon schematic of the NrsAB proteins from ICP1_2001_A (left) and NrsAB from reverted ICP1_2001_A (right). (**C**) Phage sensitivity of the strains indicated to ICP1_2001_A or reverted ICP1_2001_A (top) and quantification of the results from three biological replicates (bottom). Data are shown as the median with range. Analysis was performed using a Mann-Whitney U test (*P < 0.05).

Finally, we wanted to test whether correcting the nonsense mutation in *orf229* restores the ability of ICP1_2001_A to infect wild-type A103. To do this, we cloned a 338-bp region of the functional *orf229* from ICP1_2001 into pDL1531. After mating the plasmid into the permissive El Tor strain E7946, we passaged ICP1_2001_A twice on this strain. In doing so, we expected some phages to recombine with the plasmid, thus gaining a functional copy of *orf229* (Fig. 6A). Recombinant phages were selected on A103, and four plaques were subsequently plaque-purified and subjected to whole-genome sequencing. All four had the nonsense mutation corrected to the wild-type tyrosine in addition to reversion of G75E to the wild-type sequence in ICP1_2001. This reversion occurred spontaneously as it was just outside the region cloned into pDL1531. This indicates that this residue is important for the proper function of ORF229. These results show the importance of encoding a functional ORF228 (PRS) and ORF229 (NAPRT) for ICP1 to counter the Nezha phage defensive system in classical biotype *V. cholerae.* Based on our results and the strong homology of ORF228 and ORF229 to enzymes involved in NAD^+^ synthesis, we named the ICP1 system **N**AD^+^ **r**egenerating **s**ystem AB (*nrsAB*), where *orf228* is *nrsA* and *orf229* is *nrsB.*

### ICP1 NrsAB regenerates NAD^+^ in the presence of Nezha but imposes a fitness cost in its absence

**Fig 7:**
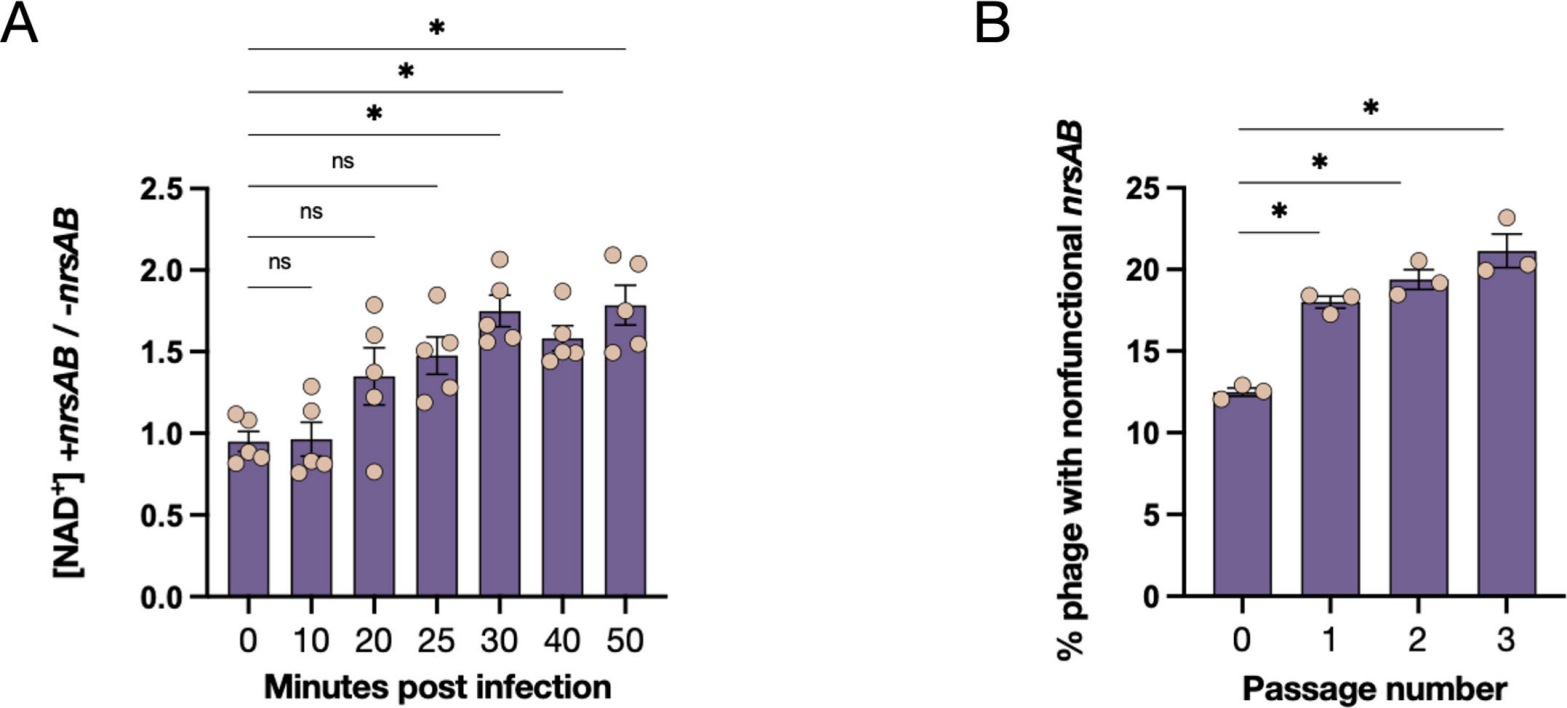
The *nrsAB* system of ICP1 regenerates NAD^+^ but imposes a fitness cost when the host lacks Nezha. (**A**) Quantification of cellular NAD^+^ levels from five biological replicates during 50 minutes of infection with ICP1_2001 or ICP1_2004_A. Data are shown as the ratio of NAD^+^ concentration +*nrsAB*/-*nrsAB*. (**B**) Competing infections of a host that lacks Nezha between two isogenic phages that either contain or lack a functional *nrsAB* system. A stop codon at position 29 of *nsrB* abolishes function and was monitored by deep sequencing of the input population and three subsequent passages performed in three biological replicates. Data are shown as the standard error of the mean. Analysis was performed using one-way ANOVA with Dunnett’s multiple comparison test (*P < 0.05).

Based on protein homology, we hypothesized that NrsA and NrsB play a role in NAD^+^ regeneration during phage infection. To test our hypothesis, we measured cellular NAD^+^ levels in WT A103 infected with ICP1_2001 (+*nrsAB*) and ICP1_2004_A (–*nrsAB*). To do this, we infected mid-exponential growth phase A103 at an MOI of 5 and isolated samples over a time course of 50 minutes post-infection. We then measured NAD^+^ levels using the Promega NAD/NADH Glo kit at t = 10, 20, 25, 30, 40, and 50 minutes post-infection. We also included t = 0 as a no-phage control. In doing so, we observed significantly higher levels of NAD^+^ in groups infected with ICP1_2001 when compared to groups infected with ICP1_2004_A (Fig. 7A). This suggests that NrsAB is indeed regenerating NAD^+^ during infection to circumvent the effects of Nezha.

Interestingly, of the 75 ICP1 isolate sequences deposited in Genbank, five lack *nrsAB,* and 60 have the Y29* mutation in *nrsB*. Functional *nrsAB* sequences are only present in four strains isolated from Bangladesh before 2002 and six strains isolated from the Congo in 2017. If *V. cholerae* in those locations at those times contained Nezha (or another NADase), there would have been a selection to maintain functional *nrsAB* genes. By contrast, although Nezha is found in some current environmental strains of *V. cholerae*, it is not found in hundreds of pathogenic strains isolated from the stools of cholera patients. Hence, for most ICP1 phages, there may be no selection to maintain functional *nrsAB* genes. Moreover, if this operon imposes a fitness cost to ICP1 by interfering with host NAD^+^ metabolism during phage infection, it would be advantageous to lose or inactivate the *nrsAB* genes. Consistent with this, while we could clone and express *nrsB* alone, we could not do so with *nrsAB,* suggesting that the intact operon is lethal.

We next tested whether a functional *nrsAB* system affects phage replication in hosts that lack Nezha. We competed WT ICP1_2001_A (inactive *nrsB* with a stop codon at position 29 of NrsB) and the reverted ICP1_2001_A (tyrosine at position 29) during infection of an El Tor host that naturally lacks Nezha. We used three independent input mixtures of ~10% WT phage and~90% reverted phage and passaged the mixture three times. The region containing position 29 of *nrsB* in the input pools and subsequent passages was amplified by PCR and subjected to deep sequencing for quantification of the two phage strains. We observed that WT ICP1 phage with a stop codon at position 29 represented 12% of the input pool and significantly increased with each passage to more than 20% by the third passage (Fig. 7B). Hence, in hosts that lack Nezha, an isogenic phage with a non-functional *nrsAB* system outcompeted a phage with an active system. We conclude that in the absence of a NADase, a functional *nrsAB* system imposes a fitness cost on phage replication.

## DISCUSSION

In the molecular arms race between bacteria and phages, many anti-phage systems have evolved, which phages evolve to inhibit or circumvent. We identified two anti-phage systems in the classical biotype *V. cholerae*, Gabija and Nezha. Gabija is a high molecular weight complex comprising multiple GajA and GajB subunits (14, 15, 27). GajA is a class 2 OLD (overcoming lysogenization defect) family DNA endonuclease, and GajB belongs to the 1A superfamily of helicase proteins. OLD family proteins were first identified in P2 phage and are classified into class 1, if they occur alone, or class 2, if they occur with a UvrD helicase protein (28–30). The role of Gabija in phage defense was first shown using a heterologous system, whereby Gabija from the Gram-positive spore former *Bacillus cereus* was expressed in *E. coli* and shown to provide resistance to T7 phage (15). Systems homologous to Gabija are present in about 15% of sequenced bacterial and archaeal genomes (13, 20, 31). Similarly, Nezha also encodes for a high molecular weight complex comprising 12 SIR2-like protein (SIR2) subunits and 6 HerA subunits. SIR2 functions as an NAD^+^ hydrolase with homology to eukaryotic sirtuin proteins (32), while HerA belongs to the helicase family (16, 17). The role of SIR2-like proteins in phage defense was also first shown using a heterologous system, whereby the Thoeris system from *B. cereus* was expressed in *Bacillus subtilis* and shown to provide resistance to several *B. subtilis* phages via NAD^+^ hydrolysis (33, 34). Subsequent studies report that bacteria encode a diverse repertoire of NAD^+^ depleting systems, including argonautes (pAGO), defense-associated sirtuins (DSR), SEF/IL-17 Receptor (SEFIR), and antiviral ATPase/NTPase of the STAND superfamily (AVAST) (19, 35, 36)

In contrast to many of the studies cited above that used heterologous systems to show the function of Gabija and Nezha in phage defense, we show their function in a natural host/phage system. Using a series of defined deletions, we showed that in classical biotype *V. cholerae*, Gabija plays a minor role in defense against ICP1 and ICP2 phages and that Nezha plays a major role in resistance to ICP1, ICP2, and ICP3. We confirmed this by mutating the active site of SIR2 to abolish NAD^+^ hydrolase activity, which resulted in a complete loss of phage resistance. Moreover, deleting the HerA helicase resulted in the loss of phage resistance. This is consistent with a previous report that the helicase subunit of Nezha is required for the NADase activity of SIR2 (16, 17).

Nezha hydrolyzes NAD^+^ to generate adenosine diphosphate ribose (ADPR) and nicotinamide (NAM), thus depleting this essential coenzyme within the host cell. In a *B. subtilis* strain engineered to express a homologous DSR2 system from another *B. subtilis* strain, NAD^+^ depletion by DSR2 blocked SPR phage replication, while phi3T phage was resistant to the effects of DSR2. phi3T encodes for an anti-DSR2 protein called DSAD1, which directly interacts with and antagonizes the NAD^+^-depleting effector (19). By contrast, we showed that phage ICP1_2001 indirectly counters the effect of Nezha by encoding two NAD^+^ regenerating proteins, NrsA and NrsB.

Based on homology, we initially assumed NrsA was a phosphoribosyl pyrophosphate synthetase, converting ribose 5-phosphate to phosphoribosyl pyrophosphate, and NrsB, a nicotinate phosphoribosyl transferase that adds nicotinic acid to phosphoribosyl pyrophosphate to generate nicotinic acid mononucleotide (37). However, during the preparation of this manuscript, a biochemical characterization of proteins highly homologous to NrsAB was deposited in bioRxiv (38). The authors reported that homologs of NrsA and NrsB are adenosine diphosphate ribose-pyrophosphate synthetases (Adps) and nicotinamide ADPR-transferases (Namat), respectively. It was shown that Adps adds a pyrophosphate (PP) to ADPR in an ATP-dependent manner to generate ADPR-PP. This molecule is then utilized by the second enzyme, Namat, to catalyze a previously uncharacterized reaction that conjugates nicotinamide onto ADPR-PP, directly generating NAD^+^. Given that ICP1 NrsA and NrsB share strong structural homology over their entire lengths with Adps and Namat, including identity across the active site residues (Fig. S5 and S6), NrsA and NrsB likely catalyze the same reactions, thus comprising an NAD^+^ regenerating system. In support of this, we observed higher levels of NAD+ when a strain with Nezha was infected by a phage that expresses the NrsAB system as compared with a phage lacking the system (Fig. 7A). However, most if not all modern *V. cholerae* El Tor strains lack Nezha and, in this context, functional NrsAB imposes a fitness cost on phage replication (Fig. 7B).

In summary, we establish that the classical biotype *V. cholerae* encodes two phage defensive systems, Gabija and Nezha, absent from El Tor biotype strains. Nezha is the more robust defense against the ICP phages. Some ICP1 phages harbor a functional NAD^+^ regenerating system to counter Nezha and likely other NADases, while the majority harbor a non-functional system that can be easily reactivated by a reversion mutation should NADases be encountered. This adds to the rapidly advancing knowledge of the diverse molecular mechanisms bacteria and phages use against each other. This is important not only for understanding the impact of phages on the life cycles and evolution of bacteria but also informs the use of phages in preventing and treating bacterial infections.

## MATERIALS AND METHODS

### Bacterial strains and growth conditions

Bacterial strains, phages, and plasmids are listed in Table S2. Plasmid sequences are provided in Files S1 to S3. Bacteria were cultured at 37°C in ysogeny broth (LB) with aeration or on LB agar. Media were supplemented with 50 µg/mL of apramycin (Apra), spectinomycin (Spec), kanamycin (Kan), or carbenicillin (Carb) or 100 µg/mL streptomycin (Sm) when appropriate. Complementation from plasmids was done by inducing gene expression with either 1 mM isopropyl-β-D-thiogalactopyranoside (IPTG) or 0.2% arabinose (Ara). The details of plasmid, phage mutant, and bacterial strain construction are provided in File S4.

### Plaque assays

Bacterial strains were grown overnight in LB at 37°C with aeration. The cultures were then back-diluted into fresh LB and grown at 37°C to an OD_600_ of 0.5. 0.1 mL of the culture was added to 6 mL of 50°C soft agarose (LB with 0.3% agarose), poured on top of LB agar plates, and allowed to cool and solidify. Phage were serially diluted 10-fold and 5 µL was spotted or 10 µL was dribbled on the overlay. Plates were incubated at 37°C for 3–4 hours, and the number of plaques counted. For NrsB complementation, A103 (pDL1530) was grown in LB supplemented with Kan to an OD_600_ of 0.5. 0.1 mL of the culture was added to 6 mL of 50°C soft agarose supplemented with Kan and Ara to maintain the plasmid and induce expression of the P_BAD_ promoter, respectively.

### Phage competition

The competition was performed with WT ICP1_2001_A and reverted ICP1_2001_A. Three independent input phage mixtures were generated with ~10% WT and ~90% reverted phage. Each input was passed through a permissive El Tor strain for a total of three passages. For this, E7946 was grown overnight in LB at 37°C and back diluted the next day into 50 mL LB. The culture was grown at 37°C with aeration to OD_600_ of 0.1 before adding phage at an MOI of 0.01. The culture was incubated for 2 hours at 37°C until full lysis was observed. Phage lysates were isolated as described in the phage propagation step without PEG treatment. The input and three passages were then PCR amplified for the region spanning the premature stop codon at position 29 of *nrsB* and deep sequencing using the Illumina platform. The percentage of reads with a stop codon at position 29 was determined using the basic variant detection tool on the CLC Genomics Workbench software (Qiagen).

### Quantification of cellular NAD^+^

We used the luciferin-based Promega NAD/NADH Glo kit (Promega, Inc) to measure NAD^+^. First, A103 was grown overnight in LB at 37°C with aeration. The next day, the culture was back-diluted into fresh LB and grown at 37°C to an OD_600_ of 0.5. Next, 1.1 mL of the culture was removed and placed in a 1.5 mL microfuge tube and 0.1 mL was removed to a separate tube for t = 0 and snap-frozen. To the remaining 1 mL, ICP1 was added at an MOI of 5, and 0.1 mL of the infected culture was immediately aliquoted into 1.5 mL microfuge tubes and incubated at 37°C with aeration. At each timepoint following infection (t = 10, 20, 25, 30, 40, and 50 minutes), samples were snap-frozen to stop the infection prior to sample processing. We optimized the manufacturer’s protocol to measure NAD^+^ by treating the snap-frozen samples with 0.1 mL bicarbonate lysis buffer [100 mM sodium carbonate, 20 mM sodium bicarbonate, 10 mM nicotinamide, 0.05% triton X-100, 1% cetyltrimethylammonium bromide (CTAB)]. After thawing the samples, 50 µL of 0.8N HCl was added and samples were heated at 60°C for 15 minutes. Then, 50 µL of 0.5N Tris-base was added to neutralize the solution before placing the samples on ice. We added 25 µL of our sample with 25 µL of the luciferin detection reagent to a white opaque 96-well plate to measure luminescence using the Biotek H1 synergy plate reader. We used an NAD^+^ standard (New England Biolabs) to generate a standard curve and extrapolate concentration from the RLU values. The standard was treated the same as the samples described above.

### Statistical analysis

Data were first log-transformed and checked for normality using the Shapiro-Wilk test. Parametric data were analyzed using the one-way ANOVA test with Dunnett’s multiple comparisons. Non-parametric data were analyzed using either the Mann-Whitney U test or the Kruskal-Wallis test with Dunn’s multiple comparisons. *P* values < 0.05 were considered as significant. GraphPad Prism software, version 10, was used for all statistical analysis.

## Data Availability

All data and plasmids are available upon request. The plasmids have been deposited to Addgene with the following ID numbers: pDL1403 (ID 226635), pDL1530 (ID 226636), and pDL1531 (ID 226637).

## SUPPLEMENTAL MATERIAL

File S1 mbio.02457-24-s0001.txt Annotated pDL1403 sequence.

File S2 mbio.02457-24-s0002.txt Annotated pDL1530 sequence.

File S3 mbio.02457-24-s0003.txt Annotated pDL1531 sequence.

File S4 mbio.02457-24-s0004.docx Plasmid, phage, and strain construction; phage propagation; whole genome sequencing.

Supplemental Figures mbio.02457-24-s0005.pdf Figures S1-S6.

Table S1 mbio.02457-24-s0006.pdf The ICP3 mutant has a frame-shift in *orf17*.

Table S2 mbio.02457-24-s0007.pdf Bacterial strains, phages, and plasmids.

ASM does not own the copyrights to Supplemental Material that may be linked to, or accessed through, an article. The authors have granted ASM a non-exclusive, world-wide license to publish the Supplemental Material files. Please contact the corresponding author directly for reuse.

## References

[1] Mutreja A, Kim DW, Thomson NR, Connor TR, Lee JH, Kariuki S, Croucher NJ, Choi SY, Harris SR, Lebens M, Niyogi SK, Kim EJ, Ramamurthy T, Chun J, Wood JLN, Clemens JD, Czerkinsky C, Nair GB, Holmgren J, Parkhill J, Dougan G. 2011. Evidence for multiple waves of global transmission within the seventh cholera pandemic. Nature 477:462–465. doi:10.1038/nature10392 21866102 PMC3736323

[2] Seed KD, Bodi KL, Kropinski AM, Ackermann H-W, Calderwood SB, Qadri F, Camilli A. 2011. Evidence of a dominant lineage of Vibrio cholerae-specific lytic bacteriophages shed by cholera patients over a 10-year period in Dhaka, Bangladesh. mBio 2:e00334-10. doi:10.1128/mBio.00334-10 21304168 PMC3037004

[3] Boyd CM, Angermeyer A, Hays SG, Barth ZK, Patel KM, Seed KD. 2021. Bacteriophage ICP1: a persistent predator of Vibrio cholerae. Annu Rev Virol 8:285–304. doi:10.1146/annurev-virology-091919-072020 34314595 PMC9040626

[4] Silva-Valenzuela CA, Camilli A. 2019. Niche adaptation limits bacteriophage predation of Vibrio cholerae in a nutrient-poor aquatic environment. Proc Natl Acad Sci U S A 116:1627–1632. doi:10.1073/pnas.1810138116 30635420 PMC6358685

[5] Seed KD, Faruque SM, Mekalanos JJ, Calderwood SB, Qadri F, Camilli A. 2012. Phase variable O antigen biosynthetic genes control expression of the major protective antigen and bacteriophage receptor in Vibrio cholerae O1. PLoS Pathog 8:e1002917. doi:10.1371/journal.ppat.1002917 23028317 PMC3441752

[6] O’Hara BJ, Barth ZK, McKitterick AC, Seed KD. 2017. A highly specific phage defense system is a conserved feature of the Vibrio cholerae mobilome. PLoS Genet 13:e1006838. doi:10.1371/journal.pgen.1006838 28594826 PMC5481146

[7] Hsueh BY, Severin GB, Elg CA, Waldron EJ, Kant A, Wessel AJ, Dover JA, Rhoades CR, Ridenhour BJ, Parent KN, Neiditch MB, Ravi J, Top EM, Waters CM. 2022. Phage defence by deaminase-mediated depletion of deoxynucleotides in bacteria. Nat Microbiol 7:1210–1220. doi:10.1038/s41564-022-01162-4 35817890 PMC9830645

[8] Boyd CM, Subramanian S, Dunham DT, Parent KN, Seed KD. 2024. A Vibrio cholerae viral satellite maximizes its spread and inhibits phage by remodeling hijacked phage coat proteins into small capsids. Elife 12:RP87611. doi:10.7554/eLife.87611 38206122 PMC10945586

[9] Seed KD, Lazinski DW, Calderwood SB, Camilli A. 2013. A bacteriophage encodes its own CRISPR/Cas adaptive response to evade host innate immunity. Nature 494:489–491. doi:10.1038/nature11927 23446421 PMC3587790

[10] Angermeyer A, Hays SG, Nguyen MHT, Johura F-T, Sultana M, Alam M, Seed KD. 2021. Evolutionary sweeps of subviral parasites and their phage host bring unique parasite variants and disappearance of a phage CRISPR-cas system. mBio 13:e0308821. doi:10.1128/mbio.03088-21 35164562 PMC8844924

[11] Bourgeois J, Lazinski DW, Camilli A. 2020. Identification of spacer and protospacer sequence requirements in the Vibrio cholerae type I-E CRISPR/Cas system. mSphere 5:e00813-20. doi:10.1128/mSphere.00813-20 33208517 PMC7677007

[12] Box AM, McGuffie MJ, O’Hara BJ, Seed KD. 2016. Functional analysis of bacteriophage immunity through a type I-E CRISPR-Cas system in Vibrio cholerae and its application in bacteriophage genome engineering. J Bacteriol 198:578–590. doi:10.1128/JB.00747-15 26598368 PMC4719448

[13] Doron S, Melamed S, Ofir G, Leavitt A, Lopatina A, Keren M, Amitai G, Sorek R. 2018. Systematic discovery of antiphage defense systems in the microbial pangenome. Science 359:eaar4120. doi:10.1126/science.aar4120 29371424 PMC6387622

[14] Antine SP, Johnson AG, Mooney SE, Leavitt A, Mayer ML, Yirmiya E, Amitai G, Sorek R, Kranzusch PJ. 2024. Structural basis of Gabija anti-phage defence and viral immune evasion. Nature 625:360–365. doi:10.1038/s41586-023-06855-2 37992757 PMC10781630

[15] Cheng R, Huang F, Wu H, Lu X, Yan Y, Yu B, Wang X, Zhu B. 2021. A nucleotide-sensing endonuclease from the Gabija bacterial defense system. Nucleic Acids Res 49:5216–5229. doi:10.1093/nar/gkab277 33885789 PMC8136825

[16] Tang D, Chen Y, Chen H, Jia T, Chen Q, Yu Y. 2023. Multiple enzymatic activities of a Sir2-HerA system cooperate for anti-phage defense. Mol Cell 83:4600–4613. doi:10.1016/j.molcel.2023.11.010 38096825

[17] Shen Z, Lin Q, Yang X-Y, Fosuah E, Fu T-M. 2023. Assembly-mediated activation of the SIR2-HerA supramolecular complex for anti-phage defense. Mol Cell 83:4586–4599. doi:10.1016/j.molcel.2023.11.007 38096827 PMC11418745

[18] Gao L, Altae-Tran H, Böhning F, Makarova KS, Segel M, Schmid-Burgk JL, Koob J, Wolf YI, Koonin EV, Zhang F. 2020. Diverse enzymatic activities mediate antiviral immunity in prokaryotes. Science 369:1077–1084. doi:10.1126/science.aba0372 32855333 PMC7985843

[19] Garb J, Lopatina A, Bernheim A, Zaremba M, Siksnys V, Melamed S, Leavitt A, Millman A, Amitai G, Sorek R. 2022. Multiple phage resistance systems inhibit infection via SIR2-dependent NAD^+^ depletion. Nat Microbiol 7:1849–1856. doi:10.1038/s41564-022-01207-8 36192536

[20] Tesson F, Hervé A, Mordret E, Touchon M, d’Humières C, Cury J, Bernheim A. 2022. Systematic and quantitative view of the antiviral arsenal of prokaryotes. Nat Commun 13:2561. doi:10.1038/s41467-022-30269-9 35538097 PMC9090908

[21] Néron B, Denise R, Coluzzi C, Touchon M, Rocha EPC, Abby SS. 2023. MacSyFinder v2: improved modelling and search engine to identify molecular systems in genomes. Peer Community J 3. doi:10.24072/pcjournal.250

[22] Tesson F, Planel R, Egorov AA, Georjon H, Vaysset H, Brancotte B, Neron B, Mordret E, Atkinson GC, Bernheim A, Cury J. 2024. A comprehensive resource for exploring antiphage defense: DefenseFinder webservice, wiki and databases. bioRxiv. doi:10.1101/2024.01.25.577194

[23] Beckman DA, Waters CM. 2023. Vibrio cholerae phage ICP3 requires O1 antigen for infection. Infect Immun 91:e0002623. doi:10.1128/iai.00026-23 37594274 PMC10501212

[24] Lim ANW, Yen M, Seed KD, Lazinski DW, Camilli A. 2021. A tail fiber protein and a receptor-binding protein mediate ICP2 bacteriophage interactions with Vibrio cholerae OmpU. J Bacteriol 203:e0014121. doi:10.1128/JB.00141-21 33875544 PMC8316142

[25] Seed KD, Yen M, Shapiro BJ, Hilaire IJ, Charles RC, Teng JE, Ivers LC, Boncy J, Harris JB, Camilli A. 2014. Evolutionary consequences of intra-patient phage predation on microbial populations. Elife 3:e03497. doi:10.7554/eLife.03497 25161196 PMC4141277

[26] Hove-Jensen B, Andersen KR, Kilstrup M, Martinussen J, Switzer RL, Willemoës M. 2017. Phosphoribosyl diphosphate (PRPP): biosynthesis, enzymology, utilization, and metabolic significance. Microbiol Mol Biol Rev 81:e00040-16. doi:10.1128/MMBR.00040-16 28031352 PMC5312242

[27] Oh H, Koo J, An SY, Hong S-H, Suh J-Y, Bae E. 2023. Structural and functional investigation of GajB protein in Gabija anti-phage defense. Nucleic Acids Res 51:11941–11951. doi:10.1093/nar/gkad951 37897358 PMC10681800

[28] Sironi G. 1969. Mutants of Escherichia coli unable to be lysogenized by the temperate bacteriophage P2. Virol (Auckl) 37:163–176. doi:10.1016/0042-6822(69)90196-2 4884707

[29] Schiltz CJ, Lee A, Partlow EA, Hosford CJ, Chappie JS. 2019. Structural characterization of Class 2 OLD family nucleases supports a two-metal catalysis mechanism for cleavage. Nucleic Acids Res 47:9448–9463. doi:10.1093/nar/gkz703 31400118 PMC6755086

[30] Schiltz CJ, Adams MC, Chappie JS. 2020. The full-length structure of Thermus scotoductus OLD defines the ATP hydrolysis properties and catalytic mechanism of Class 1 OLD family nucleases. Nucleic Acids Res 48:2762–2776. doi:10.1093/nar/gkaa059 32009148 PMC7049728

[31] Payne LJ, Todeschini TC, Wu Y, Perry BJ, Ronson CW, Fineran PC, Nobrega FL, Jackson SA. 2021. Identification and classification of antiviral defence systems in bacteria and archaea with PADLOC reveals new system types. Nucleic Acids Res 49:10868–10878. doi:10.1093/nar/gkab883 34606606 PMC8565338

[32] Brachmann CB, Sherman JM, Devine SE, Cameron EE, Pillus L, Boeke JD. 1995. The SIR2 gene family, conserved from bacteria to humans, functions in silencing, cell cycle progression, and chromosome stability. Genes Dev 9:2888–2902. doi:10.1101/gad.9.23.2888 7498786

[33] Ofir G, Herbst E, Baroz M, Cohen D, Millman A, Doron S, Tal N, Malheiro DBA, Malitsky S, Amitai G, Sorek R. 2021. Antiviral activity of bacterial TIR domains via immune signalling molecules. Nature 600:116–120. doi:10.1038/s41586-021-04098-7 34853457

[34] Ka D, Oh H, Park E, Kim J-H, Bae E. 2020. Structural and functional evidence of bacterial antiphage protection by Thoeris defense system via NAD^+^ degradation. Nat Commun 11:2816. doi:10.1038/s41467-020-16703-w 32499527 PMC7272460

[35] Zaremba M, Dakineviciene D, Golovinas E, Zagorskaitė E, Stankunas E, Lopatina A, Sorek R, Manakova E, Ruksenaite A, Silanskas A, Asmontas S, Grybauskas A, Tylenyte U, Jurgelaitis E, Grigaitis R, Timinskas K, Venclovas Č, Siksnys V. 2022. Short prokaryotic Argonautes provide defence against incoming mobile genetic elements through NAD^+^ depletion. Nat Microbiol 7:1857–1869. doi:10.1038/s41564-022-01239-0 36192537

[36] Millman A, Melamed S, Leavitt A, Doron S, Bernheim A, Hör J, Garb J, Bechon N, Brandis A, Lopatina A, Ofir G, Hochhauser D, Stokar-Avihail A, Tal N, Sharir S, Voichek M, Erez Z, Ferrer JLM, Dar D, Kacen A, Amitai G, Sorek R. 2022. An expanded arsenal of immune systems that protect bacteria from phages. Cell Host Microbe 30:1556–1569. doi:10.1016/j.chom.2022.09.017 36302390

[37] Canto C. 2022. NAD^+^ precursors: a questionable redundancy. Metabolites 12:630. doi:10.3390/metabo12070630 35888754 PMC9316858

[38] Osterman I, Samra H, Rousset F, Loseva E, Itkin M, Malitsky S, Yirmiya E, Millman A, Sorek R. 2024. Phages reconstitute NAD^+^ to counter bacterial immunity. bioRxiv. doi:10.1101/2024.02.11.579819 39322677

