# Supplementary material for: A Vibrio cholerae anti-phage system depletes nicotinamide adenine dinucleotide to restrict virulent bacteriophages
Source: mBio. 2024 Oct 8;15(11):e02457-24. doi: 10.1128/mbio.02457-24 (PMC11559045; doi:10.1128/mbio.02457-24)
Supplement: File S4 — Plasmid, phage, and strain construction; phage propagation; whole genome sequencing. [file mbio.02457-24-s0004.docx]

**File S4. Plasmid, phage, and strain construction; Phage propagation; Whole genome sequencing.**

A diaminopimelic acid (DAP) auxotrophic Pir-expressing RP4 conjugation donor derivative of *E. coli* TG1, called TG1 donor pir, was constructed in two steps. First, we used P1 to transduce the RP4 mating function genes flanked by Apramycin- and Zeocin-resistance genes from *E. coli* MFDpir (1) into TG1 to create TG1 donor. TG1 donor pir was then created using lambda-red recombineering with a PCR fragment amplified from MFDpir in which the *dapA* gene was replaced with those that express Pir and erythromycin resistance. TG1 donor pir strains were supplemented with 0.5 mM DAP. Plasmids pDL1530 and pDL1531 were constructed for this study to allow inducible expression of cloned genes and for allelic exchange, respectively. Both have a p15a origin of replication and an RP4 oriT for conjugal transfer. Their complete annotated sequences are provided in **File S2 and 3**.

**Construction of *V. cholerae* mutants.** Marked deletion and point mutation constructs were made by splicing-by-overlap extension (SOE) PCR, and then the linear dsDNA products were transformed into naturally competent *V. cholerae*. Natural competence was induced by growth overnight in 0.7% (7 g/L) Instant Ocean salts (Aquarium Systems) containing shrimp chitin flakes (Sigma-Aldrich) at 30˚C without aeration. Transformants were selected on LB agar supplemented with the appropriate antibiotic(s).

**Transduction of PLE2.** Natural competence was used to insert the *aad9* gene flanked by promoter and terminators downstream of and in the same direction as the *orf2* gene in PLE2. The resulting strain was then infected with ICP1_2001 at a MOI of 1. After 2 hrs of incubation at 37˚C, the infected culture was centrifuged, and the supernatant was filtered. The recipient A103 was grown overnight at 37˚C in LB. The next day, 0.1 mL of the overnight culture was infected with 10 µL of the filtrate and the volume was adjusted to 1 mL by addition of 0.89 mL of LB. The culture was incubated for 1 hr at 37˚C and plated on LB agar supplemented with Spectinomycin to select for transductants.

**Bacterial conjugation.** Chemically competent *E. coli* TG1 donor pir cells were first transformed with the plasmids listed in Table S2. Next, plasmids were moved into *V. cholerae* by mating with TG1 donor pir cells. Briefly, donor and recipient cells were cultured overnight in LB supplemented with DAP and Carbenicillin or LB alone at 37˚C, respectively. The next day, 0.5 ml of donor and recipient were pelleted and washed twice with LB before resuspending in 50 µL of LB. A 1:1 mixture was added to a sterile 0.2 µm filter (Millipore) on an LB plate supplemented with DAP, the liquid allowed to soak into the underlying plate, and incubated at 37˚C for 3 hrs. Cells were recovered from the filter by vortexing in 1 ml LB. Serial dilutions were plated on LB agar supplemented with Carbenicillin to select for the exconjugates.

**Generating recombinant phage.** E7946 was grown overnight in LB at 37˚C and back diluted the next day by adding 0.1 mL into seven culture tubes each containing 10 mL LB. The culture tubes were incubated at 37˚C for 20 mins with aeration and then co-infected with a 1:1 mixture of ICP1_2001 and ICP1_2004_A at an MOI of 5. After incubating at 37˚C for 2 hrs, the infected culture was centrifuged at 10,000 RCF for 15 mins and the supernatant was filtered. Recombinant phage were isolated by plaquing on A103 PLE2::*aad9*. Plaques were plaque-purified three times and then used to generate high titer phage stocks using E7946. Phage DNA was isolated from a portion of the high titer stocks for whole genome sequencing.

**Phage propagation.** ICP1, ICP2, and ICP3 phages (Table 2) were those originally isolated from cholera patient rice-water stool samples in Dhaka, Bangladesh (2). All phage mutants constructed in this study were plaque-purified three times. High titer stocks were made by infecting mid-exponential growth phase cultures of *V. cholerae* E7946 or derivatives grown in LB at 37˚C at an MOI of 0.01 and then incubating the cultures for 2-4 hrs at 37˚C with aeration. Infected cultures were chilled to 4˚C, centrifuged at 10,000 RCF for 15 mins at 4˚C, and the supernatants were filter-sterilized through 0.45 µm bottle-top filters. Phages were precipitated by adding 25% volume of a 5x stock of polyethylene glycol (20% PEG 8000, 2.5M NaCl), mixing thoroughly, and incubating overnight at 4˚C. The precipitated phages were centrifuged at 15,000 RCF for 10 mins at 4˚C, and the supernatant removed. The phage pellets were resuspended in phage 80 buffer (0.1mM MgSO_4_, 0.1M Tris-HCl pH 7.4, 85.6mM NaCl) or 0.7% Instant Ocean salts.

**Whole genome sequencing.** Genomic DNA was extracted from bacteria or phage using the Zymo Quick DNA miniprep kit (Zymo Research) or the Promega Wizard kit (Promega), respectively, following the manufacturer’s protocol. Most bacterial and phage samples were sequenced using single or paired-end sequencing on the Illumina Nextseq platform (Illumina, Inc). ICP3 mutants were sequenced using Plasmidsaurus (Plasmidsauraus, Inc).

**REFERENCES**

1. Ferrières L, Hémery G, Nham T, Guérout A-M, Mazel D, Beloin C, Ghigo J-M. 2010. Silent mischief: bacteriophage Mu insertions contaminate products of *Escherichia coli* random mutagenesis performed using suicidal transposon delivery plasmids mobilized by broad-host-range RP4 conjugative machinery. J Bacteriol 192:6418–6427. <https://doi.org/10.1128/JB.00621-10>.
2. Seed KD, Bodi KL, Kropinski AM, Ackermann H-W, Calderwood SB, Qadri F, Camilli A. 2011. Evidence of a dominant lineage of *Vibrio cholerae*-specific lytic bacteriophages shed by cholera patients over a 10-year period in Dhaka, Bangladesh. mBio 2:e00334-10. https://doi.org/10.1128/mBio.00334-10
