# Supplementary material for: A Vibrio cholerae anti-phage system depletes nicotinamide adenine dinucleotide to restrict virulent bacteriophages
Source: mBio. 2024 Oct 8;15(11):e02457-24. doi: 10.1128/mbio.02457-24 (PMC11559045; doi:10.1128/mbio.02457-24)
Supplement: Supplemental Figures — Figures S1-S6. [file mbio.02457-24-s0005.pdf]

A

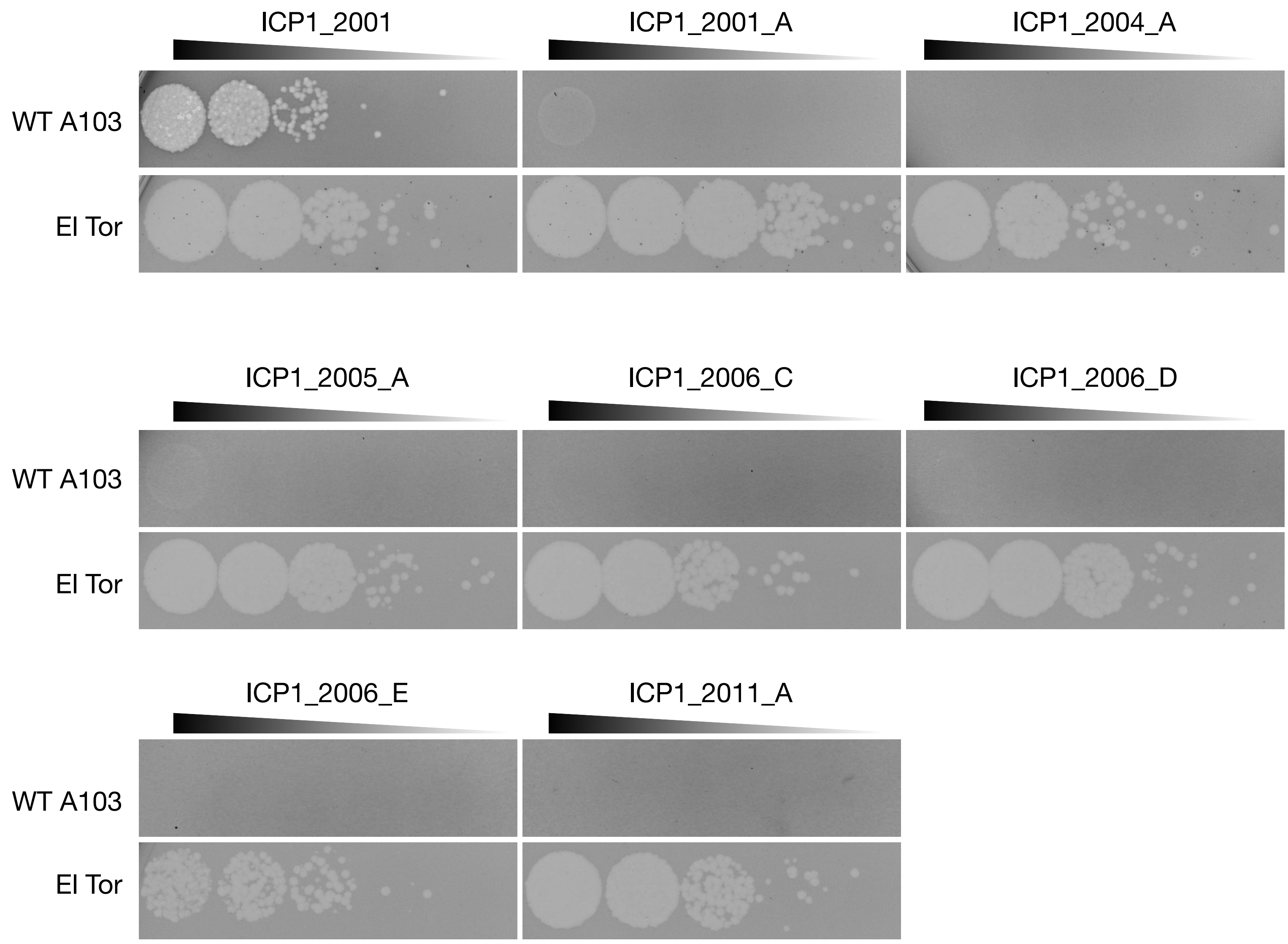

Figure S1. A103 is resistant to several ICP1 isolates except for ICP1_2001. (A) Phage sensitivity of classical biotype strain A103 (top) and El Tor strain E7946 (bottom) against eight different ICP1 phage isolates.

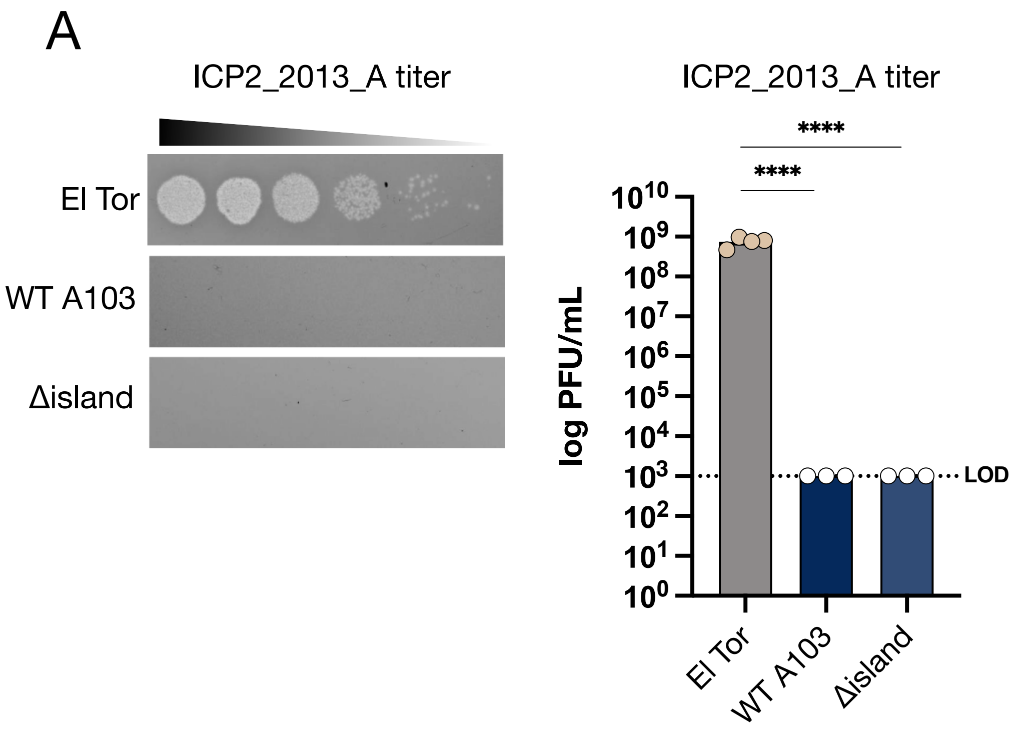

Figure S2. A103 and its derivative Δisland are resistant to ICP2 due to an OmpU mutation. (A) Phage sensitivity of the strains indicated to phage ICP2_2013_A (left) and quantification of the results from 3-4 biological replicates (right). El Tor is strain E7946; the rest are classical strain A103 wild type and derivatives. The two classical strains have a mutated OmpU receptor which prevents WT ICP2 infection. Data are shown as the standard error of the mean. Analysis was performed using one-way ANOVA with Dunnett's multiple comparison test (*$*P<0.05$, $**P<0.01$, $***P<0.001$, $****P<0.0001$*).

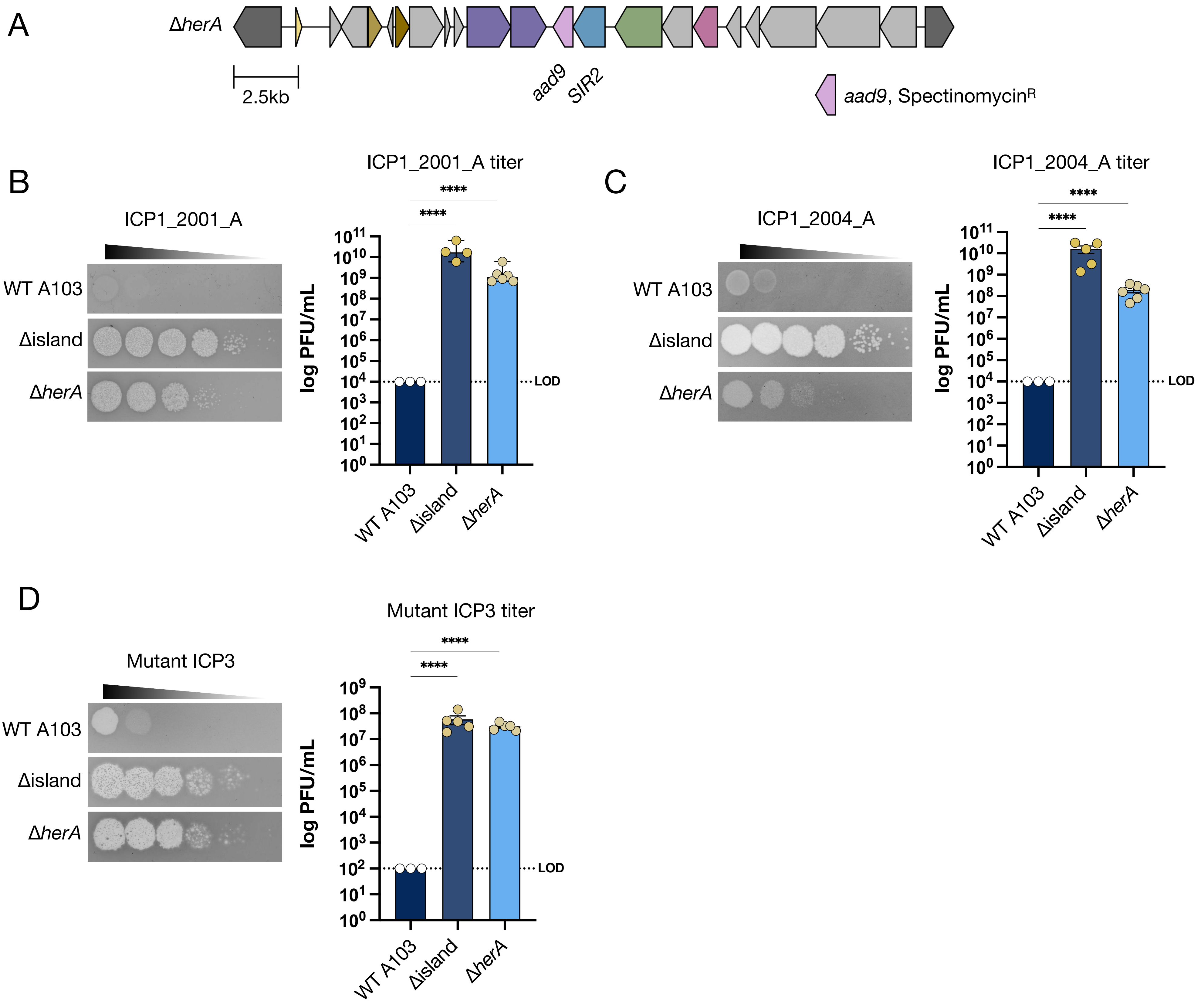

Figure S3. Deletion of herA abolishes immunity to ICP1, ICP2, and ICP3. (A) Schematic of the ΔherA deletion construct, where *herA* was replaced with a spectinomycin-resistance cassette (pink). **(B & C)** Phage sensitivity of the strains indicated to ICP1 phages (left) and quantification of the results from 3-5 biological replicates (right). **(D)** Phage sensitivity of the strains indicated to ICP3 phage (left) and quantification of the results from 3-5 biological replicates (right). Data are shown as the standard error of the mean for normally distributed data or as the median with range for non-normally distributed data. Data are shown as the standard error of the mean. Data were analyzed using one-way ANOVA with Dunnett's multiple comparison test (**P<0.05, **P<0.01, ***P<0.001, ****P<0.0001*).

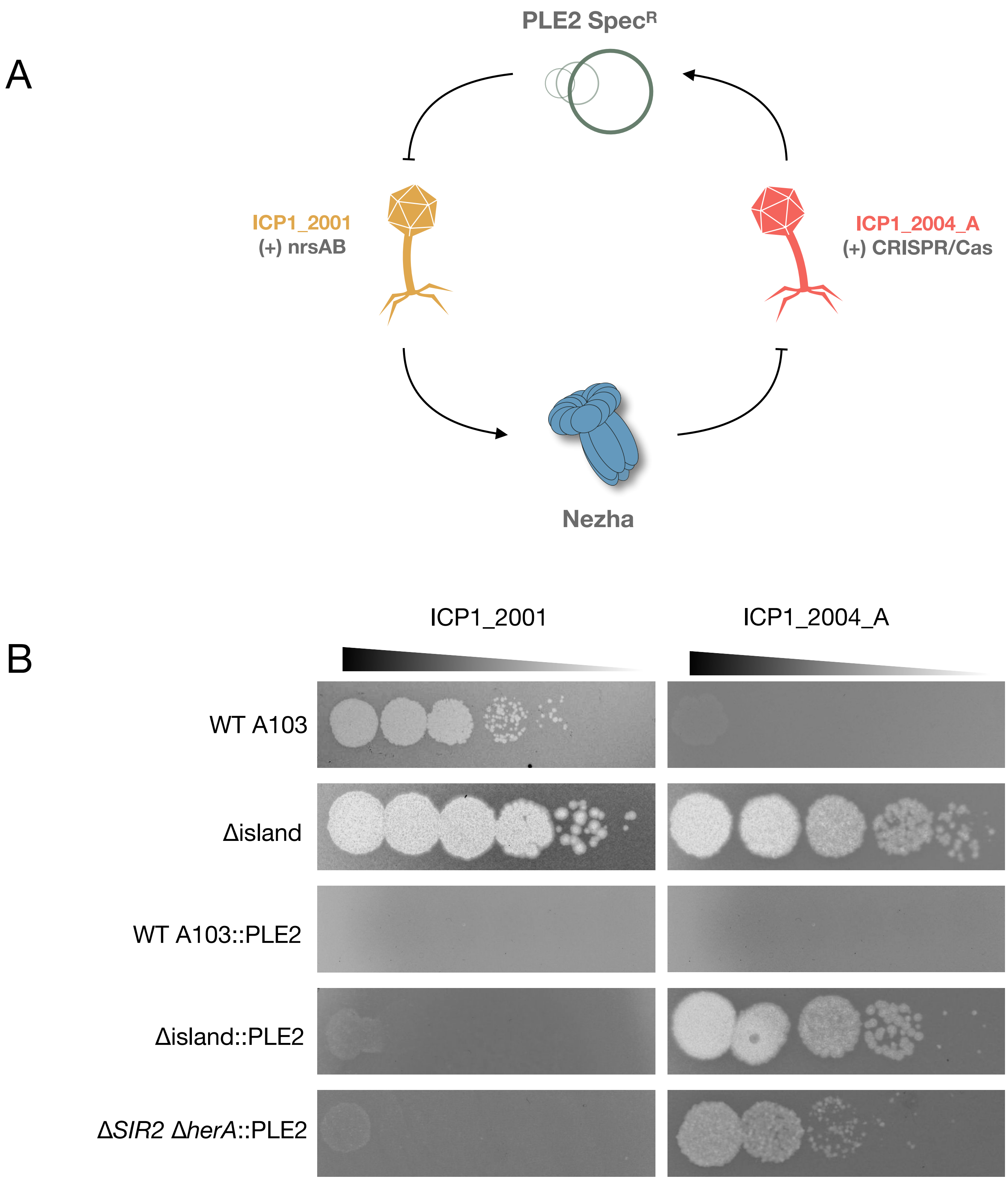

Figure S4. PLE2 antagonizes ICP1_2001 but not ICP1_2004_A.
(A) Cartoon diagram depicting differential sensitivities of ICP1_2001 and 2004_A to PLE2 marked with Spec^R or Nezha. Arrows indicate resistance to PLE2 or Nezha and blunt arrows indicate sensitivity to PLE2 or Nezha. **(B)** Phage sensitivity of classical biotype strain A103 and its derivatives containing PLE2 against ICP1_2001 or ICP1_2004_A. PLE2 is a potent inhibitor of ICP1_2001, but not ICP1_2004_A, which contains a CRISPR/Cas system that targets PLE2.

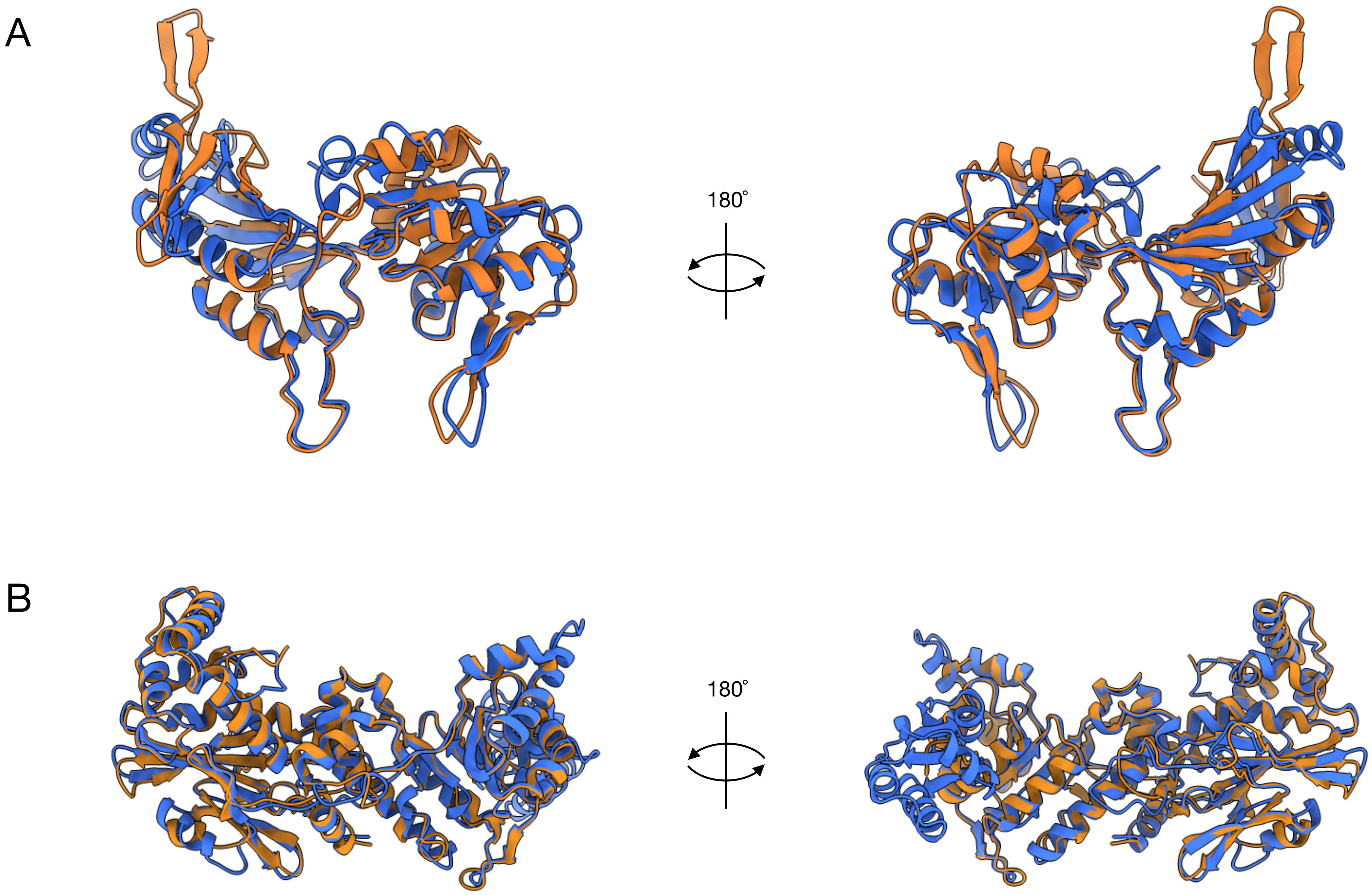

Figure S5: Structural alignment of ICP1 and Bas63 NAD^+ regenerating proteins.

A) Alpha fold protein prediction of ICP1_2001 NrsA (orange) overlayed with Bas63 Adps (blue). The structures are rotated 180˚ to visualize the proteins from the front (left) and back (right).

B) Alpha fold protein prediction of ICP1_2001 NrsB (orange) overlayed with Bas63 Namat (blue). The structures are rotated 180˚ to visualize the proteins from the front (left) and back (right).

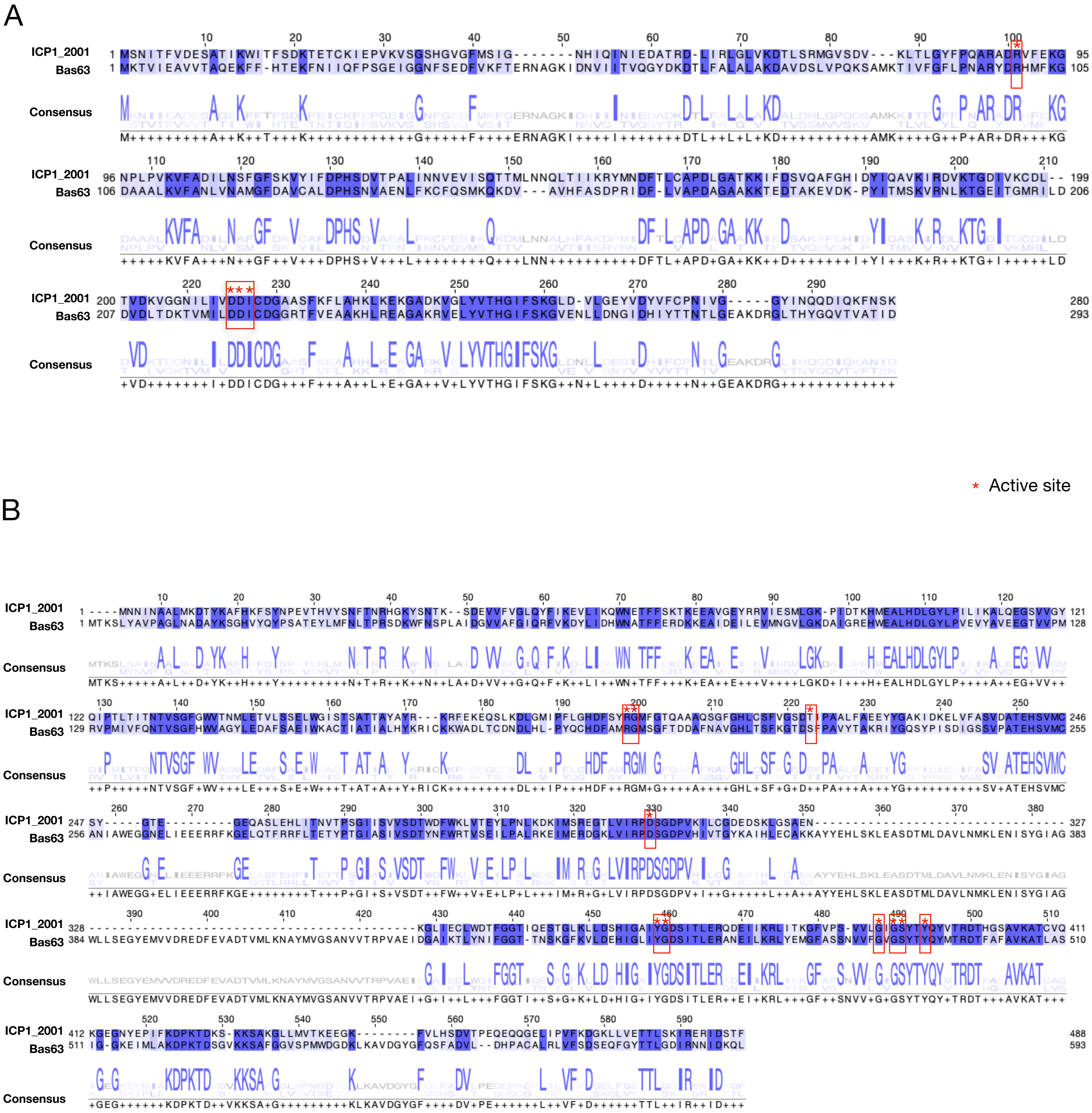

Figure S6: Comparing the ICP1_2001 NrsA and NrsB with homologues from *E. coli* phage Bas63

A) Protein alignment using the MUSCLE algorithm of ICP1_2001 NrsA (top) and Bas63 Adps (bottom). Red stars indicate active site residues that are conserved between the two proteins.

B) Protein alignment using the MUSCLE algorithm of ICP1_2001 NrsB (top) and Bas63 Namat (bottom). Red stars indicate active site residues that are conserved between the two proteins.
