# Supplementary material for: A Vibrio cholerae anti-phage system depletes nicotinamide adenine dinucleotide to restrict virulent bacteriophages
Source: mBio. 2024 Oct 8;15(11):e02457-24. doi: 10.1128/mbio.02457-24 (PMC11559045; doi:10.1128/mbio.02457-24)
Supplement: Table S1 — The ICP3 mutant has a frame-shift in orf17. [file mbio.02457-24-s0006.pdf]

Table S1. The ICP3 mutant phage has a frame-shift mutation in *orf17*.

Accession #	Gene annotation	Coding region change	Amino acid change
**NC_015159**	Mutant 1:*orf17*	YP_004251260.1: c.74del T	YP_004251260.1: p.Phe26 fs(frame shift)
	Mutant 2:*orf17*	YP_004251260.1: c.74del T	YP_004251260.1: p.Phe26 fs(frame shift)
	Mutant 3:*orf17*	YP_004251260.1: c.130del A	YP_004251260.1: p.Thr45 fs(frame shift)
	Mutant 4:*orf17*	YP_004251260.1: c.130del A	YP_004251260.1: p.Thr45 fs(frame shift)
