# Supplementary material for: A Vibrio cholerae anti-phage system depletes nicotinamide adenine dinucleotide to restrict virulent bacteriophages
Source: mBio. 2024 Oct 8;15(11):e02457-24. doi: 10.1128/mbio.02457-24 (PMC11559045; doi:10.1128/mbio.02457-24)
Supplement: Table S2 — Bacterial strains, phages, and plasmids. [file mbio.02457-24-s0007.pdf]

Supplementary Table 2. Bacterial strains, phages, and plasmids used.

***V. cholerae* strains**	**Description**	**Source, Accession numbers**
**E7946**	El Tor biotype, serogroup O1 strain, Sm^R	(1), CP024162, CP024163
**A103**	Classical biotype, serogroup O1, Inaba strain A103 N.I 1990, Sm^S	(2), GCA_001254575.1
**7607**	A103 PLE2::*aad9*, Spec^R	This study
**7856**	A103 Δisland*::aac(3)-Iva,* PLE2::*aad9*, Spec^R	This study
**7857**	A103 *ΔherA Δsir2::aac(3)-Iva,* PLE2::*aad9*, Spec^R	This study
**7616**	A103 (pDL1403), Carb^R	This study
**7675**	A103 Δisland*::aac(3)-IVa*, Apra^R	This study
**7722**	A103 *ΔherA Δsir2::aac(3)-IVa*, Apra^R	This study
**7723**	A103 *ΔgajA ΔgajB::aac(3)-IVa*, Apra^R	This study
**7724**	A103 *ΔgajA ΔgajB ΔherA Δsir2::aac(3)-IVa*, Apra^R	This study
**7697**	A103 *herA sir2::aac(3)-Iva ,* Apra^R	This study
**7774**	A103 *ΔompU*:: E7946 *ompU::aad9,* Spec^R	This study
**7775**	A103 Δisland*::aac(3)-Iva, ΔompU*::E7946 *ompU::aad9*, Apra^R, Spec^R	This study
**7776**	A103 *ΔgajA ΔgajB::aac(3)-Iva, ΔompU*::E7946 *ompU*, Apra^R, Spec^R	This study
**7777**	A103 *ΔherA Δsir2::aac(3)-Iva, ΔompU*::E7946 *ompU*, Apra^R, Spec^R	This study
**7778**	A103 *ΔgajA ΔgajB ΔherA Δsir2::aac(3)-Iva, ΔompU*::E7946 *ompU*, Apra^R, Spec^R	This study
**7867**	A103 *herA sir2::aac(3)-Iva*, *ΔompU* ::E7946 *ompU ,* Apra^R , Spec^R	This study
**7858**	A103 *ΔgajA ΔgajB::aac(3)-IVa sir2*(N168A), *ΔompU* ::E7946 *ompU* , Apra^R , Spec^R	This study
**7859**	A103 *ΔgajA ΔgajB::aac(3)-IVa sir2*(H227A), *ΔompU* ::E7946 *ompU* , Apra^R , Spec^R	This study
**7792**	A103 *ΔgajA ΔgajB::aac(3)-IVa sir2*(N168A), Apra^R	This study
**7793**	A103 *ΔgajA ΔgajB::aac(3)-IVa sir2*(H227A), Apra^R	This study
**7794**	A103 *ΔherA*::*aad9*, Spec^R	This study
***E. coli* strains**		

**7401**	TG1 donor Pir, DAP auxotroph, Zeo^R, $Apra^R$, Erm^R	This study
**7611**	TG1 donor Pir, DAP auxotroph (pDL1403), $Carb^R$, Zeo^R, $Apra^R$, Erm^R	This study
**7772**	TG1 donor Pir, DAP auxotroph (pDL1530), Kan^R, Zeo^R, $Apra^R$, Erm^R	This study
**7773**	TG1 donor Pir, DAP auxotroph (pDL1531), Kan^R, Zeo^R, $Apra^R$, Erm^R	This study
**Phages**		
**ICP1_2001**	CRISPR/Cas (-), *nrsAB* active	(3), NC_015157
**ICP1_2001_A**	CRISPR/Cas (-), *nrsAB* inactive	(3), HQ641353
**ICP1_2004_A**	CRISPR/Cas (+), No *nrsAB*	(3), HQ641354
**ICP1_2005_A**	CRISPR/Cas (+), *nrsAB* inactive	(3), HQ641352
**ICP1_2006_C**	CRISPR/Cas (-), No *nrsAB*	(3), HQ641349
**ICP1_2006_D**	CRISPR/Cas (-), No *nrsAB*	(3), HQ641348
**ICP1_2006_E**	CRISPR/Cas (+), No *nrsAB*	(4), MH310934
**ICP1_2011_A**	CRISPR/Cas (+), *nrsAB* inactive	(4), MH310933
**ICP2_2013_A_Haiti**		(5), NC_024791
**ICP3**		(2), NC_015159
**Plasmids**		
**pDL1403**	Expression vector for *tfox* and *qstR*. p15a *oriR*, RP4 *oriT*, pTac, and *bla*	This study

**pDL1530**	Expression vector for ICP1 *nrsB*. p15a *oriR*, RP4 *oriT*, pBad, and *apH*(*3*)	This study
**pDL1531**	Vector for allelic exchange. p15a *oriR*, RP4 *oriT*, pBad, and *apH*(3)	This study

References

1. Levine MM, Black RE, Clements ML, Cisneros L, Saah A, Nalin DR, Gill DM, Craig JP, Young CR, Ristaino P. 1982. The Pathogenicity of Nonenterotoxigenic *Vibrio cholerae* Serogroup 01 Biotype El Tor Isolated from Sewage Water in Brazil. J Infect Dis 145:296–299. https://doi.org/10.1093/infdis/145.3.296
2. Mutreja A, Kim DW, Thomson N, Connor TR, Lee JH, Kariuki S, Croucher NJ, Choi SY, Harris SR, Lebens M, Niyogi SK, Kim EJ, Ramamurthy T, Chun J, Wood JL, Clemens JD, Czerkinsky C, Nair GB, Holmgren J, Parkhill J, Dougan G. 2011.Evidence for multiple waves of global transmission within the seventh cholera pandemic. Nature.477(7365):462–5. https://doi.org/10.1038/nature10392
3. Seed KD, Bodi KL, Kropinski AM, Ackermann H-W, Calderwood SB, Qadri F, Camilli A. 2011. Evidence of a Dominant Lineage of *Vibrio cholerae*-Specific Lytic Bacteriophages Shed by Cholera Patients over a 10-Year Period in Dhaka, Bangladesh. mBio 2:e00334-10. https://doi.org/10.1128/mBio.00334-10
4. Angermeyer A, Das MM, Singh DV, Seed KD. 2018. Analysis of 19 Highly Conserved *Vibrio cholera*e Bacteriophages Isolated from Environmental and Patient Sources Over a Twelve-Year Period. Viruses 10:299. https://doi.org/10.3390/v10060299
5. Seed KD, Yen M, Shapiro BJ, Hilaire IJ, Charles RC, Teng JE, Ivers LC, Boncy J, Harris JB, Camilli A. 2014. Evolutionary consequences of intra-patient phage predation on microbial populations. eLife 3:e03497. https://doi.org/10.7554/eLife.03497
